# Stochastic Reliability-Based Design Optimization Framework for the Steel Plate Girder with Corrugated Web Subjected to Corrosion

**DOI:** 10.3390/ma15207170

**Published:** 2022-10-14

**Authors:** Damian Sokołowski, Marcin Kamiński

**Affiliations:** Chair of Structural Reliability, Faculty of Civil Engineering, Architecture and Environmental Engineering, Lodz University of Technology, Al. Politechniki 6, 90-924 Lodz, Poland

**Keywords:** reliability-based design optimization, stochastic perturbation technique, Monte Carlo simulation, semi-analytical method, topological optimization, corrugated web, corrosion

## Abstract

This paper proposes the framework for reliability-based design optimization (RBDO) of structural elements with an example based on the corrugated web I-girder. It tackles the problem of topological optimization of corroding structures with uncertainties. Engineering restrictions follow a concept of the limit states (LS) and extend it for stability and eigenfrequency assessment. The reliability constraints include all the LS; they are computed according to first- and second-order reliability methods. The RBDO example minimizes the bridge girder cross-section while satisfying the structural reliability level for the ultimate and the serviceability limit states, stability, and eigenfrequency. It takes into consideration two uncorrelated random effects, i.e., manufacturing imperfection and corrosion. They are both Gaussian; the first of them is applied at assembly time, while the second is applied according to the time series. The example confronts three independent FEM models with an increasing level of detailing, and compares RBDO results for three concurrent probabilistic methods, i.e., the iterative stochastic perturbation technique (ISPT), the semi-analytical method, and the Monte Carlo simulation. This study proves that the RBDO analysis is feasible even for computationally demanding structures, can support automation of structural design, and that the level of detailing in the FEM models influences its results. Finally, it exemplifies that reliability restrictions for LS are much more rigorous than for their deterministic counterparts, and that the fastest ISPT method is sufficiently accurate for probabilistic calculations in this RBDO.

## 1. Introduction

Contemporary structural designs require powerful tools for optimization purposes, which must be effective, fast, and easy to use. Together with an exponential increase in computational power, the traditional analytical approach to the optimization of Civil Engineering structures has become significantly outdated. In the majority of designs, this approach has already been replaced by more accurate deterministic methods, among which the finite element method plays a crucial role, and they are largely implemented in commercial software. Such an optimization strategy is applied for example in [1]; its goal is commonly focused on structural topology [2,3]. The traditional deterministic design appears to be suboptimal when significant uncertainties must be taken into account, such as climatic loads, material uncertainties, assembly errors, and corrosion or soil conditions, just to name a few. They cannot be avoided in structural and especially civil engineering designs. This is why a new concept called reliability-based design optimization (RBDO) arose, where the uncertainties are directly included in the design. An acceptable contrast between the efficiency of deterministic and reliability optimization is given in [4] and an exhaustive review of the concepts of RBDO is available [5] or in [6]. RBDO is well researched, especially for steel truss structures e.g., [7,8], and frames e.g., [9,10], where the computation effort of each optimization loop is acceptable. They are not so common in more complex problems involving the plate, shell, or composite structures, where the computation is much more demanding and the amount of strength and serviceability checks much higher; such construction is considered in this study. This extreme computational intensiveness is well depicted in [11] and still constitutes a major issue. Computational intensiveness is caused not only by the number of limit states but also by the high number of deterministic and random design variables, whose impact on these states is not always known a priori to RBDO. This aspect is commonly tackled by an initial sensitivity analysis, which determines the susceptibility of structural response to a variation of design parameters. It has been used with success for many years e.g., in [12], and eliminates unnecessary variables from further computation. Application of the RBDO could also include corrosion effects [13], which constitute a major topic of this paper.

Although some new concepts are still being put forward [14,15], the RBDO methods are quite mature now and allow a very efficient (but computationally demanding) design. What they still lack is the ability to define a reliable life of structures, so that the optimization is not aimed purely at modifications of the pristine materials, but also takes into consideration the degradation of its work throughout the service life. This is what we propose in our concept of RBDO, which allows a service life optimization with a reliability-based design approach for the determination of the reliable service life of steel structures that are subject to corrosion. Corrosion affects the strength and serviceability of steel structures, including their capacity, stability, and durability. Its effects are depicted for example in [16,17]. It is also a major reason for the careful and costly maintenance of steel structures [18] and together with fire softening it constitutes the main weakness of this material. Corrosion affects steel already at erection time, which is quite different from reinforced concrete where the onset of corrosion is shifted from this initial time [19] and calculated for example according to Fick’s second law. This is because steel structures are directly affected by chloride attack and are not covered by other materials. Of course, there exist a variety of covers such as special paints or chrome plating and various additives (see for instance weathering steel), but even such techniques do not prevent this phenomenon completely; they require repetitive application during the service life of steel constructions. Corrosion especially affects thin-walled structures, where small pitting corrosion placed in a susceptible place, or a small reduction in thickness may cause serious loss of capacity. A fine example of such structures are those with sinusoidally corrugated webs currently applied in girders (also arched ones [20]) and also composite structures, where the deck is made of concrete and the web of constructional steel [21].

The corrugated web considerably increases rigidity and shear capacity [22], and decreases sensitivity to a local stability loss in the web [23], thus reducing the occurrence of local buckling. It also allows for reduced self-weight [24], as compared to conventional flat web I-girders, and does not increase the complexity of execution, as trusses do. The usage of these assets has increased, especially in structural applications such as bridges, pedestrian walkways, hangars, and industrial buildings. Interestingly, corrugated webs also outperform flat webs in energy dissipation and could be used in anti-seismic structures [25]. The first studies of the SIN web girders were conducted in the late 1990s [26], yet they are still new in civil engineering applications and have several disadvantages for such applications. They offer a weaker contribution to bending [27] and cause an additional normal stress distribution in flanges coming from transverse bending [28] compared to the regular I-beams; they also have complex bending–shear interaction [29]. To make matters worse, there still is a lack of design standards or specifications dealing with the behavior of such webs and SIN web girders.

This is why in recent years there has been intensive research on their basic behavior connected with stress [30], elastic critical moment, buckling [31,32], and shear [33,34]; they are computed in this study within the concept of limit states. A very interesting problem is the capacity and serviceability of such constructions in fire conditions [35], which considerably reduce the bearing capacity of steel structures and also their reliability. 

Reliability-based computations of structural elements with a corrugated web are also available [36,37] and even a weight optimization could be found [38], but RBDO has not yet been proposed for such structures; this is especially true when corrosion is the leading random effect. Consequently, there is a need to develop recommendations that properly address the reliability issues of such girders, which is also the reason for their choice as an example for the proposed framework.

The principal objective of this paper is to propose an RBDO framework with a special focus on structural elements and constructions that must satisfy complex restrictions of engineering standards. The secondary goal is to successfully apply this framework to a computationally challenging example of the sinusoidally corrugated web I-girder. The study assumes the possibility of optimizing the topology of civil engineering structures subjected to uncertain corrosion evolution and engineering restrictions for a specified reliable service life. Further, it assumes that FEM modeling accuracy and probabilistic solver play a crucial role in the accuracy and timing of RBDO results. Next, it assumes the importance of the steel and environment type as well as the choice of the WLSM weighing function for the solution. 

Therefore, a design optimization framework and additional theoretical background for time-dependent reliability civil engineering analysis have been presented and applied for the case study of the steel plate girder with a sinusoidally corrugated I web. The results obtained in this paper would allow for more optimal designing of such structures and may be applied to other steel and concrete structures.

## 2. Theoretical Background

**Figure 1:**
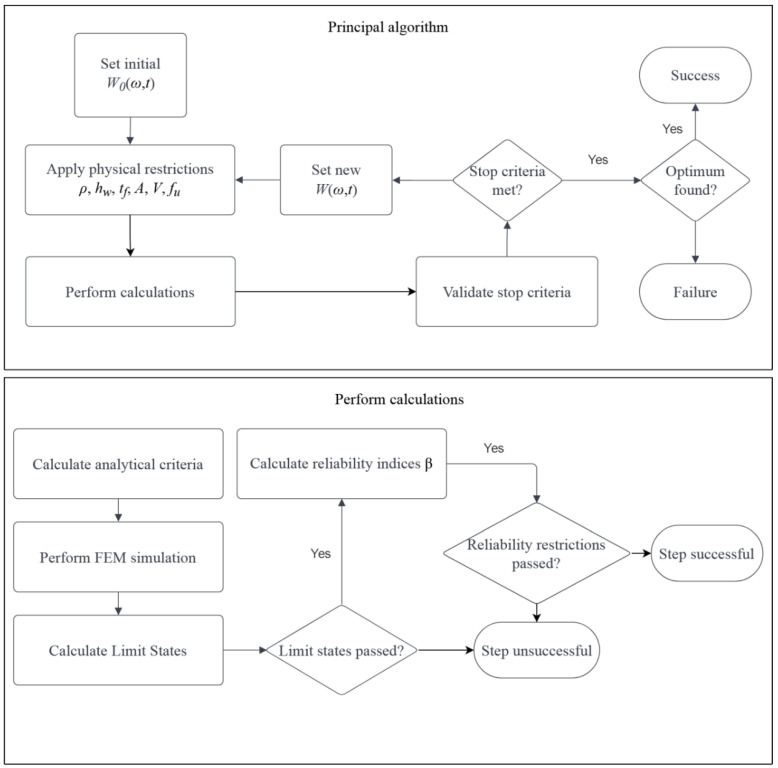
Principal algorithm of the method and calculation process.

According to the current design codes, the structural elements’ durability period adopted is 50 years, and this is usually ensured by repeated design trials with the goal of minimizing weight or cost, alternatively optimization of its capacity for different limit states. In such terms, a designer solves an optimization problem with a clearly defined set of restrictions, which are first of all (1) the minimum capacity of the element, secondly (2) geometrical constraints, (3) material restrictions, (4) maximum deformations, and (5) other physical constraints. The most tedious work is usually required in the first of these, i.e., in ensuring a minimum capacity of the element. This is because the element (and an entire structure) is subjected to multiple loads of different morphology and with different placement (wind, snow, traffic load, vibrations, self-weight, machinery, etc.), which may or may not occur at the same time. That is why an engineer must check the structural capacity for multiple combinations, which almost always require a different computational approach. This work proposes a framework, according to which such optimization could be utilized with a specified goal of optimization. It takes into consideration a classical approach of limit states proposed by the Eurocode 0 [39] and also a more refined, higher-order probabilistic design method directly using the reliability theory, where the judgment of service life is done based on the reliability indices $\beta_{g}$ of the designed elements. Such refinement is advised in Appendices B and C of this design norm [39]. Within this framework, multiple structural elements may be assessed, and the entire structure could be optimized. The objective function may be purely topological, as in the below example, but it may also be cost-oriented, when the total cost of an element is optimized, or may minimize the difference of all the indices $\beta_{g}$. The method selected for optimization purposes is deterministic non-gradient regular search, in which the main loop encompasses the subsequent steps of (1) deterministic verification of restrictions, (2) verification of the given limit states, and finally, (3) checks of the reliability indices. This is shown in Figure 1, where the optimization problem depends on the design time t and input uncertainty ω; at each step, a new topology $W(\bar{\omega };t)$ is proposed. A non-gradient approach is selected because in most civil engineering designs gradient cannot be explicitly computed. This is because each of the combinations defined in multiple limit states would be different and movement in any direction of the design domain may give converse results for multiple combinations. The main objective of this algorithm is structural optimization and the main loop depicted in Figure 1 starts with an arbitrary point in the optimization domain (starting geometry of the element) that should be selected according to the engineering practice. At first, the most straightforward conditions are checked, i.e., geometrical, material, and physical constraints. Then, a cross-sectional class is determined, because its change demands different checks of the limit states and the reliability indices. Upon this, the finite element method is utilized to solve the mechanical problem and its results are used in the determination of both, the limit states and reliability indices. Verification of the physical restrictions and cross-sectional class is straightforward and depends strictly on the selected material, geometry, and static scheme of the specific element. The FEM must be carefully formulated for each part so that the parameters existent in the objective function could be easily (or preferably automatically) ameliorated and the subsequent model solved; this will be explained in detail for the specific context of the example.

**Figure 2:**
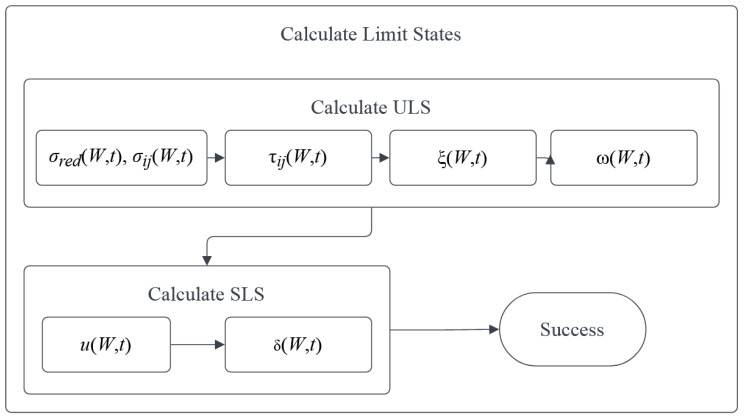
Procedure for the determination of the limit states.

Designing processes requiring a detailed explanation include the ‘Calculate Limit States’, ‘Calculate Reliability indices’ $\beta_{g}$, and ‘Validate stop criteria’. According to a common definition, a limit state is a state in which the construction element or an entire structure will fail due to a specified external action. The design codes in their basic form define the ultimate limit state (ULS) and serviceability limit state (SLS), which describe either the conditions in which the construction will fail (ULS) or will stop working in an acceptable way (SLS); the hierarchy of LS is shown on Figure 2. If the design is made based on the FEM, not the analytical approach, each element must meet five fundamental conditions for the linear regime of the structural materials. First, (1) the general stresses $\sigma_{ij}$ and (2) the reduced (commonly Huber–Mises) stresses $\sigma_{red}$ must be lower than the plastic limit. In the case of the linear civil engineering structures, these are limited to the longitudinal normal stresses $\sigma_{11}$ and the shear stresses $\sigma_{12}$ (or τ). Additionally, (3) the structure must be stable and buckle neither locally nor globally (but local buckling is sometimes permitted when additional structural elements are added to the designed part); this constraint is depicted in Figure 2 by ξ (W,t). The last condition in the ULS encompasses (4) the first eigenfrequencies, which must be high enough not to be triggered by the wind or traffic; usually, the minimum is set to 5 Hz. In the SLS, (5) deformations of this element must be limited, which include the deflections u (W,t) and also displacements at its ends (or borders) and connections to other parts $\delta_{i}\;(W,t)$.

One may also include requirements for exceptional states, such as collision, fire loads, or checks for fatigue, but they are out of the scope of this work; this is important because of their rare inclusion in design purposes in civil engineering practice. All the mentioned states could be checked implicitly using the results of the subsequent FEM analyses, which are static, eigenfrequency, and critical load tasks available in the most common programs such as ABAQUS, ANSYS, Catia, or DIANA. Please note, that some of the limit states may be checked globally for each part or an entire structure, such as ξ (W,t) or ϖ (W,t), while the others must be met for all the points of the structure (in FEM, for all the elements). These include the stresses, strains, deflections, deformations, and displacements, all of them depending strictly on the objective function—the topology of the element. If all restrictions in both limit states are met, the optimization could proceed to the next process—a check of the reliability indices $\beta_{g}$.

**Figure 3:**
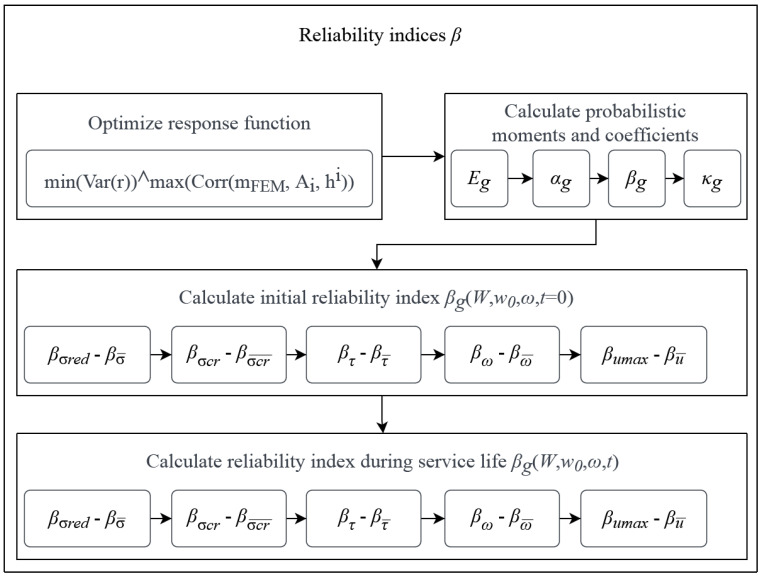
Procedure for the determination of reliability indices.

A process of the determination of reliability indices $\beta_{g}$ serves for a final check of the restrictions and could be started only if all the previous ones are met. This is because the limit functions *g* are almost directly taken from the limit states and used in the numerator of the reliability indices $\beta_{g}$. This process is depicted in Figure 3, which defines the flow of computations at this stage of optimization. It starts with optimization of the response function, then it determines the probabilistic coefficients, and finally calculates the reliability indices serving as the final restrictions. These indices are computed first at time *t* = 0 with an initial uncertainty $\omega_{0}$ and only then for *t*$\;\epsilon \;\left({0;t}_{d}\right)$ and ω, where *t_d_* is designed service life. This is because the design may be already unacceptable when no aging (or corrosion) of the material is included. The checks of reliability are performed for all the restrictions from the limit states separately and each of them must be met so that the current topology can be accepted. In this process, neither the amount nor the probability density function (PDF) is strictly limited, and the total number of uncertainty sources depends upon the probabilistic methods applied, as does the PDF for each of these.

Checks for t=0 are performed for both the first-order reliability method (FORM) and the second-order reliability method (SORM) in sequential order; this order of analysis is defined by O in Figure 3. The last computation of reliable service life is computed solely by the FORM (for O=1) and plotted in the service time domain. Unlike the majority of papers that propose only one method of probabilistic calculus (see for example [40]), we propose three concurrent methods, the iterative stochastic perturbation technique, the semi-analytical method (AM), and the crude Monte Carlo simulation (MCS). Please note that the computation of reliability restrictions is not limited to the above methods.

Determination of the response function, the first task in reliability-focused computations, serves as an inner optimization problem. It is solved at each optimization step just before the determination of probabilistic characteristics. It is devoted to the determination of a continuous response function of the capacity of the girder $f_{c}\left(W\right)$ from its discrete representation solved via the FEM. This is required in computations of subsequent limit functions g defined as the difference of this capacity and the reaction to external action or an engineering limit *f_e_*(*W*).

(1) $$g(W)=f_{c}(W)-f_{e}^{}(W).\;$$

Please note that the analytical formula relating the objective function and the functions of girder capacity cannot be explicitly derived analytically, which is why the FEM is used for its retrieval. The inner optimization problem is solved by deterministic non-gradient search in the discrete domain of the order of the response polynomial and the number of terms in this polynomial. Restrictions include several terms $n_{A}{}_{i}\;>\;0\;\epsilon \;N$ and the order of polynomial $P_{O}\;>\;0\;\epsilon \;N$. The optimization aim is twofold—minimization of the weighted least squares method (WLSM) variance and maximization of correlation of the polynomial and the FEM results.

(2) $$\min\left(Var\left(\bar{r}\right)\right)\wedge \max\left(Corr\left(m_{FEM},A_{i}h^{i}\right)\right)$$

where higher precedence is set to the correlation. The stop function is generally not required in this problem because of a finite number of allowable points in the discrete optimization domain. $P_{O}$ is limited to 10 ÷ 30, firstly because of no real correlation gain for higher order polynomials, and secondly because of difficulties with its behavior outside or neighboring to the probing range of the FEM. Such optimization is performed for each of the limit functions. In the exemplary problem, only $u_{\max}$ was taken into consideration, whose an explicit mathematical formulation is proposed as

(3) $$\begin{array}{l} u\left(\omega ;t\right)=N\cdot q\left(\omega ;t\right)=N\cdot A_{i}(t)\cdot h^{i}\left(\omega ;t\right);\;\;\;r^{2}={\left(m_{FEM}-A_{i}h^{i}\right)}^{2}:\;\;\;\min\left(\sum_{j=1}^{n}r_{j}^{2}\right)\to A_{j};\\ \;\;\;\;\;\;\;\;\;\;\;\;\;\;\;\;\;\;\;\;\;\;\;\;\;\min\left(Var\left(r\right)\right)\wedge \max\left(Corr\left(m_{FEM},A_{i}h^{i}\right)\right) \end{array}$$

where *u*(*ω;t*) is the maximum deflection, N stands for shape function, $A_{i}$ are defined as the coefficients of approximating polynomial, $h^{i}$ define the subsequent powers of the design variable, and r is a residuum coming from a difference of the FEM result $m_{\mathrm{FEM}}$ and the result coming from the polynomial response function $A_{i}h^{i}$.

The weighted least squares method (WLSM) solved at each optimization step uses the following polynomial approximation:

(4) $$u\left(b\right)≅D^{\left(P_{o}\right)}b^{P_{o}}=f\left(D,b\right)\;\;\;\;P_{o}=1,\ldots ,s;s<n.$$

where the polynomial basis of the *s*th order $P_{O}$ is used and solved around the web thickness of the current optimization step. This web thickness also serves as a mean value of the main random parameter included in the probabilistic calculus, indexed here by *b*. As a result *n* different pairs $\left(b^{\left(\alpha \right)},u^{\left(\alpha \right)}\right)$ for *α* = 1,…, *n* are returned, whose arguments belong to the neighborhood of expectation of b itself. The residuals in each trial point are introduced to get an algebraic condition for these expansion coefficients. They are next minimized. After relevant modifications, the following regular matrix equations are obtained

(5) $$\left({\left(J\right)}^{T}w\;J\right)\;D={\left(J\right)}^{T}\;w\;u$$

Such a system of equations (with the dimensions *n x s*) is solved symbolically in MAPLE [41]. 

**Figure 4:**
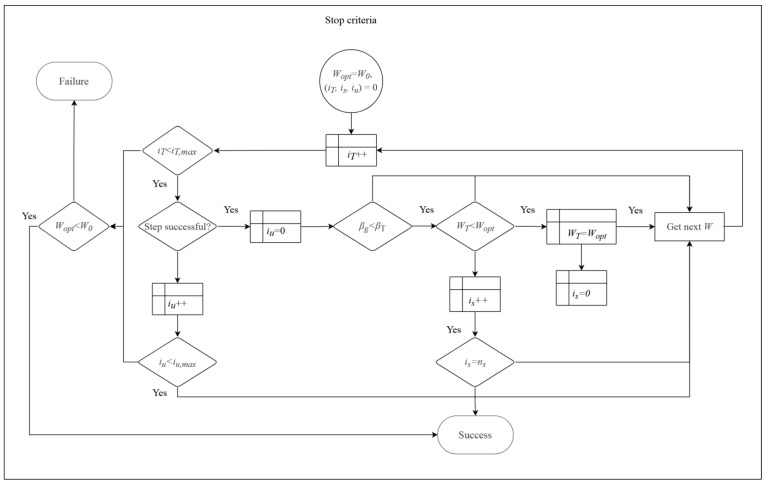
Definition of stop criteria.

The last process in the design loop is a stop condition and it is depicted in Figure 4. It evaluates if the optimized solution is found or not. Success occurs when at least one step finds a more optimal solution than $W_{0}$. Failure is identified when (1) one of the indices from restrictions is within the margin of $\beta_{T}$ at design service time $t_{d}$ and (2) $n_{s}$ subsequent steps do not decrease the objective function (fail to optimize the W). An additional stop is defined for $i_{T,max}=1000$ steps to ensure a cutoff of the optimization with weak correlation; its fulfillment may lead either to optimization success when at least one of the previous W fulfilled all restrictions or optimization failure. A more elaborate stop condition may also be applied, but its inclusion would increase the computation time, which proves critical for engineering purposes. Please note that the solution and optimization convergence will depend on the starting structural configuration $W_{0}$ and it is recommended to repeat optimization with different $W_{0}$, using a different kind of initial cross-section for example.

The reliability calculus at the initial time is only according to initial imperfections. A formulation of such a problem could be found in [42]. The final reliable life check is performed according to two random variables, namely the corrosion penetration depth and fabrication imperfection. This is possible in the stochastic context with the introduction of the relevant resulting functions of corrosion penetration depth into both, the web thickness mean value and its initial variation. Expected value of random web thickness *b* can be computed as

(6) $$E\left[b\right]=\int_{-\infty }^{+\infty }b\;p_{b}(x)dx\equiv \frac{1}{M}\sum_{i=1}^{M}b^{\left(i\right)}$$

while its variance as

(7) $$\begin{array}{l} Var\left(b\right)=\int_{-\infty }^{+\infty }{\left(b-E[b]\right)}^{2}p_{b}(x)dx+Var\left(d\right)-2Corr\left(b,d\right)\\ \;\;\;\;\;\;\;\;\;\;\equiv \frac{1}{M-1}\sum_{i=1}^{M}{\left(b^{\left(i\right)}-E[b]\right)}^{2}+Var\left(d\right)-2Corr\left(b,d\right) \end{array}$$

where *E*[*d*] is the expected value of corrosion penetration depth directly affecting the mean value of web thickness *b*; the correlation between these two stochastic variables Corr(b,d) is set to 0.

The reliability indices *β_FORM_* and *β_SORM_* may serve for initial reliability as well as structural health monitoring. The first of these could be computed in the following way:

(8) $$\beta_{g,\;\mathrm{FORM}}=\frac{E\left[g\right]}{\sigma \left[g\right]}$$

where $\beta_{g}$ stands for the reliability index of a specific limit function, *E*[g] is the expectation of this function *g*, and σ[g] is its standard deviation. A reliability index $\beta_{g,FORM}$ assumes a normal probability distribution of a given random (response) function; $\beta_{g,SORM}$ is defined as [37]

(9) $$\beta_{g,\;\mathrm{SORM}}=-\Phi^{-1}\left(P_{f2}\right)$$

where $P_{f2}$ denotes the probability of failure for the chosen probability distribution of the function relative to $\beta_{g,\;\mathrm{FORM}}$ in the following manner:

(10) $$P_{f2}=\frac{\Phi \left(\beta_{g,\;\mathrm{FORM}}\right)}{\;\sqrt{{1+\beta }_{g,\;\mathrm{FORM}\cdot }\kappa }},$$

where κ is the curvature approximating the primary surface defined by the following formula:

(11) $$\kappa =\frac{\frac{{\mathrm{du}}^{\left(m\right)}}{{\mathrm{db}}_{0}^{k_{2}}}}{{\left(1+{\left(\frac{{\mathrm{du}}^{\left(m\right)}}{{\mathrm{db}}_{0}^{k}}\right)}^{2}\right)}^{\frac{3}{2}}}.$$

## 3. Numerical Illustration

**Figure 5:**
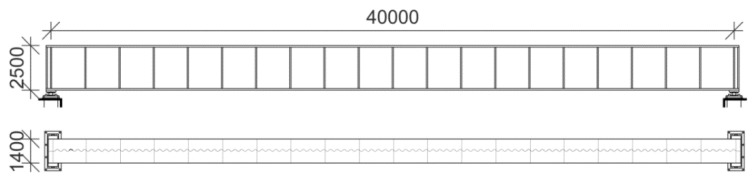
The layout of the girder in [mm].

Let us consider a corrugated web I-beam girder that is suspected of corrosion, as in Figure 5. 

In such girders, the web is predominantly affected by this phenomenon, which leads to loss of bearing capacity during its service life. This girder is subjected to topological optimization, whose goal is to optimize the cross-section that ensures reliability for 50 years of service according to Eurocode 0, Appendix B [39]. Optimization is performed within the framework proposed in Figure 1, where the limit states are checked based on three FEM models; the indices $\beta_{g}$ are verified for stresses, deflections, eigenfrequencies, and stabilities, while the final constraints of reliability are based on displacement; the following objective function *W* (ϖ;t) is proposed:

(12) $$\begin{array}{l} W\left(\omega ;\omega_{0};t\right)=\left(A_{w}\left(\omega ;\omega_{0};t\right)+2A_{f}\right)\cdot L=\left(t_{w}\left(\omega ;\omega_{0};t\right)\cdot h_{w}+2A_{f}\right)\cdot L\\ \;\;\;\;\;\;\;\;\;\;\;\;\;\;\;=\left(\left(t_{w_{0}}\left(\omega_{0}\right)-2\cdot \left(A\left(\omega \right)+t^{B\left(\omega \right)}\right)\right)\cdot h_{w}+2A_{f}\right)\cdot L \end{array}$$

As a function of $\varpi_{0}$—the coefficient of variation of the web thickness at time t=0, ϖ—coefficient of variation of corrosion as well as of time t; t=0 stands for an assembly time, in which exposure to the external environment begins. In this function, $A_{w}$ and $A_{f}$ are the cross-sectional areas of the web and the flange, $h_{w}$ stands for the height of the web, and $t_{w}$ denotes the thickness of the web. Let us note that in common civil engineering designs the flanges are much thicker than the web and are placed horizontally so that they are not so susceptible to corrosion. This is why the time dependence of this topology is generally based on the web, whose thickness decreases with corrosion. This thickness is uncertain already after its fabrication process, which is imposed by an initial coefficient of variation $\varpi_{0}$ and is then subjected to stochastic corrosion of the form $A\;\left(\varpi \right){+t}^{B\left(\varpi \right)}.$ In this term $t_{w}{=t}_{w0}\left(\omega_{0}\right)-2\cdot \left(A\left(\omega \right){+t}^{B\left(\varpi \right)}\right),$ where the quotient 2 depicts the susceptibility of $t_{w}$ to corrosion from both sides. The corrosion function strictly depends on the environment and steel type, to which it is subjected, and is taken from Melchers 2002. Its coefficients are shown in Table 1, which summarizes the corrosion parameters for rural, urban, and marine environments separately for the carbon and weathering steels. This table shows that the marine environment is specified by the highest CoV, and has expectations a little smaller than the urban environment, while the rural environment is the least invasive. The weathering steel is much less affected by corrosion, as both parameters, A and B, have smaller mean values and CVS than the carbon steel in a corresponding environment type. Nonetheless, the downside of this type of steel is its cost.

A corrosion model applied in this work comes from the additional experiments reported in the literature and is applied with the parameters adjacent to the carbon steel in an urban environment. Its expected value reads

(13) $$E\left[D\left(t\right)\right]=3.52\cdot 10^{-3}e^{2.81\cdot 10^{-24}{\left(1.05\cdot 10^{12\;}+1.00\cdot 10^{11}\ln\left(t\right)\right)}^{2}}$$

The variance is introduced as

(14) $$Var\left(D\left(t\right)\right)=7.57\cdot 10^{-3}t^{1.19}e^{1.12\cdot 10^{-1}\ln{\left(t\right)}^{2}}-5.65\cdot 10^{-4}t^{1.19}e^{5.63\cdot 10^{-2}\ln{\left(t\right)}^{2}+3.13}\;+1.24\cdot 10^{-5}t^{1.19}e^{6.24+5.63\cdot 10^{-2}\ln{\left(t\right)}^{2}}.$$

They are both truncated by the third vital number for the reader’s convenience.

The principal restrictions for this objective function come solely from the requirements of the bearing capacity, stability, eigenfrequency, and allowable deformation of the girder, which are a cross-section within its design service life. This, in turn, is defined in the current civil engineering design code, Eurocode 0 [39], which in its annexes proposes the limits of reliability index $\beta_{g}$ for each of the limit states (LS). They are divided into the ultimate limit state (ULS), in which the girder must withstand the normal, reduced, and shear stresses, have high enough first eigenfrequency, as well as not be susceptible to buckling. The second LS is the serviceability limit state (SLS), in which the deflection of this girder must be limited by the value of l/250. In such terms, there exist six restrictions for its reliability:

(15) $$\begin{array}{l} \beta_{\tau }\left(t\right)-\beta_{\hat{\tau }}\left(t\right)\geq 0;\;\beta_{\sigma_{red}}\left(t\right)-\beta_{\hat{\sigma }}\left(t\right)\geq 0;\;\beta_{\Omega }\left(t\right)-\beta_{\hat{\Omega }}\left(t\right)\geq 0;\;\\ \beta_{\sigma_{cr}}\left(t\right)-\beta_{{\hat{\sigma }}_{cr}}\left(t\right)\geq 0;\;\beta_{u_{\max}}\left(t\right)-\beta_{\hat{u}}\left(t\right)\geq 0;\;\beta_{\xi }\left(t\right)-\beta_{\hat{\xi }}\left(t\right)\geq 0;\; \end{array}$$

Further restrictions are connected to the geometry of the girder, its volume V, and cross-sectional area A, which must be all positive.

(16) $$\varrho {,\;h}_{w\;}{,\;t}_{w}{,\;t}_{f}{,\;h}_{f},\;A,\;V\;>\;0$$

The geometry of this girder includes the height of the web $h_{w\;}$, its width $t_{w}$ as well as the height $h_{f}$ and width $t_{f}$ of the flange. Let us note that the resulting checks of bearing capacity required by the Eurocode changes together with an increase of cross-sectional class and therefore its slenderness must be limited to keep the checks unified in terms of both, the flange and the web ${0\;<\;h}_{w\;}/t_{w}\;<\;72,\;0\;<\left(\frac{h_{f\;}}{2}-t_{w}-a\right)/t_{f}\;<\;9.$ Otherwise, the procedure of determination of the limit states and the reliability indices would have to be changed substantially each time the section class changes.

The material selected for the design purposes is constructional steel. This choice determines all the material restrictions, including the density $\varrho \in \;7.75–8.05{\;[g/\mathrm{cm}}^{3}]$ and the plastic limit of this steel $f_{u}$, which is here narrowed to the most common steels available on the international market, i.e., $f_{u}\in \;\left\{195,\;235,\;275,\;355,\;420,\;460\right\}\;\mathrm{MPa}$ with corresponding Young modulus E=210 GPa and Poisson ratio of μ=0.3. The material model applied in all the computations for constructional steel is linear with plastic limit $f_{u}$.

Additional restrictions proceed directly from the external requirements or the investor and include static schemes and loads. These are the designed length of the girder L=40 m, external load in form of a uniform pressure applied on the upper flange q=150 kN/m, and degrees of freedom restricted at the ends of the girder as simple supports—although they are not directly included in the objective function but in the FEM model.

The inner optimization problem is solved here with the order of response polynomial in the range of ${16\;>\;P}_{O}\;>\;0\;\epsilon \;N$. A maximum polynomial order is set, because previous optimization problems show that the solution starts to degrade already at $P_{0}\;>\;10$.

A shortened version of the results for one of these optimizations devoted to maximum deflection is given in Table 2. It shows that the accuracy of the WLSM approximation measured by its total error $E_{WLSM}$, variance $\alpha_{WLSM}$, and correlation coefficient of the response function and the discrete FEM results $C_{WLSM}$ does not necessarily increase with an increase of the polynomial order or number of terms included. Moreover, the optimum order is not possible to determine a priori to solving the optimization problem. Interestingly, the limitation of the terms with a constant $P_{O}$ has a minor influence on $C_{WLSM}$ but increases both $E_{WLSM}$ and $\alpha_{WLSM}$. It must be noted that together with an increase of the $P_{O}$ and $n_{A}$ the computation complexity and length are also increased. Generally, the optimum order is in the range of $P_{O}\in \;\left\{5;\;10\right\}$ and full polynomials are preferred. An additional problem in this inner optimization is the type of weights in the WLSM $W_{S}$. The considered weighting schemes are equal $W_{SE}$, triangular $W_{ST}$, and Dirac $W_{SD}$; the last weighting scheme places greater importance on the realizations around the mean (or middle) of the uncertain parameter, and the equal weighting scheme puts the same weight on all the discrete data points but has problems when they are not the smooth and triangular type of weights, which decreases the importance of the data points with an increase of their distance from the mean. It is quite efficient for the low $P_{O}$ but for this weighting scheme $E_{WLSM}$ and $\alpha_{WLSM}$ increase very fast together with an increase in $P_{O}$. Dirac-type of weight puts the same importance on the mean as for all the other data points. It ensures the best $C_{WLSM}$ at the highest order, keeps a very small error and low variance for a high span of $P_{O}$, and returns the smoothest approximation. This is why it was selected for further optimizations and the other weighting schemes were removed from the checks of a reliable life prediction. From Table 2, it could be also concluded that the optimum approximation was reached for a full polynomial of the ninth order and WLSM with a Dirac-type of weighting.

Such an optimized third-order maximum deflection function valid for the SLS is given below:

(17) $$E\left[R_{\mathrm{SLS}}\right]=8.09\;-\;3.13\cdot 10^{-2}\left(E\left[t_{w}\right]\right)+2.91\cdot 10^{-4}{\left(E\left[t_{w}\right]\right)}^{2}-9.71\cdot 10^{-7}{\left(E\left[t_{w}\right]\right)}^{3}.$$

In this expression, the expectation of web thickness includes an influence of fabrication error $E\left[t_{w0}\right]$ existent at t=0 and corrosion E[D(t)] so that $E\left[t_{w}\right]=E\left[t_{w0}\right]-2\cdot E\left[D\left(t\right)\right]$. Let us note that together with the total allowed deflection this maximum deflection function serves as a numerator in an expression of $\beta_{g,\;FORM}$ as $E\left[g_{\mathrm{SLS}}\right]=L/350-E\left[R_{\mathrm{SLS}}\right]$.

A variance of each limit state is available simply as $\mathrm{diff}{\left(E\left[g\right]{,t}_{w}\right)}^{2}\cdot \mathrm{Var}\left(t_{w}\right)$, where E[g] represents the expected value of each limit state; for SLS it is $E\left[g_{\mathrm{SLS}}\right]$. The variance of web thickness could be obtained as *Var*$\left(t_{w}\right)=$*Var*$\left(t_{w0}\right)+2\cdot$*Var*(D(t)); fabrication error and corrosion phenomenon are considered here as uncorrelated. An initial coefficient of variation of fabrication error is assumed as *α*$\left(t_{w0}\right)=0.05$.

The indices $\beta_{\overset{ʌ}{\sigma }}$, $\beta_{\overset{ʌ}{\sigma_{cr}}}$, $\beta_{\hat{u}}$, $\beta_{\hat{\tau }}$ and $\beta_{\hat{\Omega }}$ define the threshold for each of the reliability restrictions, while $\beta_{\sigma_{\mathrm{red}}}{,\;\beta }_{u_{\max}}{,\;\beta }_{\Omega }{,\;\beta }_{\tau }$, and $\beta_{\sigma_{cr}}$ denote the indices of reliability computed according to the first-order reliability method (FORM, see Equation (8)) or second-order reliability method (SORM, see Equation (9)). They strictly follow the types of checks made in the limit states of the framework but include the uncertainty disregarded in LS; a detailed formulation for a FORM and SORM is given in the theoretical background. Restrictions of $\beta_{g}$ are always in the form of a difference between the threshold indices $\beta_{\overset{ʌ}{g}}$ and the resulting indices defining the girder $\beta_{g}$. The threshold indices are piecewise constant functions with required service time${\;t}_{s}$, which by default is 50 years [39]. The minimum values corresponding to $t_{s}=50$ for $\beta_{\overset{ʌ}{\sigma }}$, $\beta_{\overset{ʌ}{\sigma_{cr}}}$, $\beta_{\hat{\tau }}$ = 3.8 and $\beta_{\hat{u}}$, $\beta_{\hat{\Omega }}$, $\beta_{\hat{\xi }}$ = 1.5. This is basically because the former defines the ULS and the latter the SLS. In its first-order formulation, $\beta_{g}$ is a simple quotient of the expected value of the limit function g − E[g] and the standard deviation of g − *σ*[g]. In turn, the limit function is a difference between the capacity of the girder and its response to an external action or an engineering limit; there may exist multiple limit functions for a single engineering structure (such as for this girder). Owing to this, there exist also multiple formulations of the limiting indices, whose definition is very close to the limit states existent in the approach of Eurocode; for the context of this example, the following indices must be defined:

- $\beta_{\sigma }$—a reliability index for the maximum normal stress;
- $\beta_{\sigma_{\mathrm{red}}}$—for the maximum stress according to the Huber–Mises criterion;
- $\beta_{\tau }$—for the ultimate shear stress;
- $\beta_{u_{\max}}$—for the ultimate deflection of this girder;
- $\beta_{\Omega }$—for the eigenfrequency;
- $\beta_{\xi }$—for the stability defined by the critical load (CL).

In this example $\beta_{T}=0.3$, $i_{s}=1$, $i_{u}=5$, and $i_{T}=100$. Additionally, the process of determination of $\beta_{g}$ was postponed until the limit states were optimized, which allowed substantial optimization time savings.

### 3.1. Numerical Model Description

The FEM simulations are provided by the use of an FEM system ABAQUS [44] with the use of three 3D full-scale models reported below:

- Volumetric (model 1)—having the highest level of detailing including the ribs and welds, made with hexa—end tetrahedral elements;
- Shell model with ribs (model 2)—with a moderate level of detailing including inner and support ribs, based on the quad-dominated shell elements;
- Shell model without ribs (model 3)—with only the basic level of detailing including solely the web and flanges, based on the quad-dominated shell elements.

**Figure 6:**
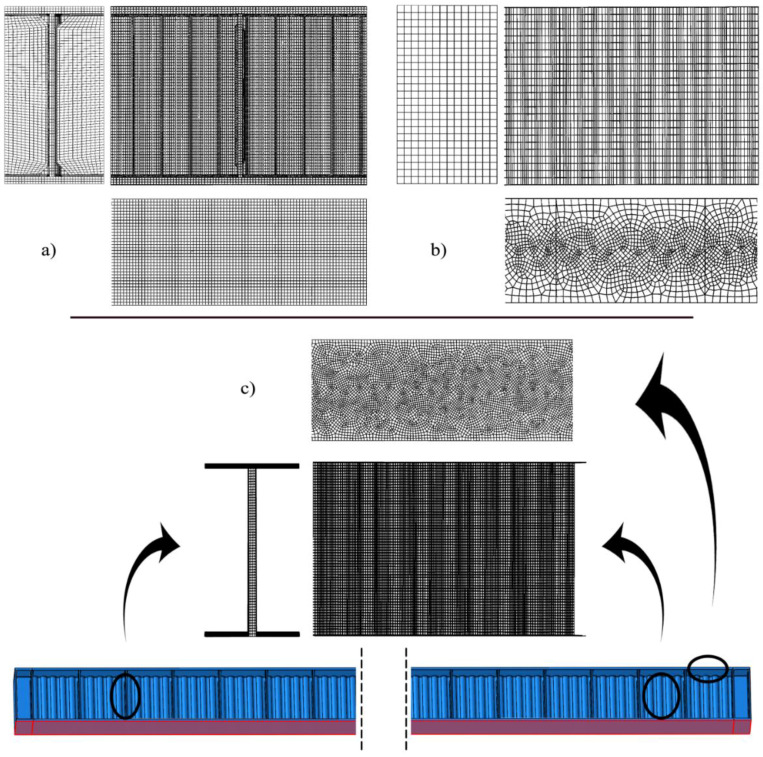
ABAQUS discretization of the girder, (**a**) with ribs and welds (volumetric, model 1), (**b**) with ribs (shell, model 2), (**c**) without ribs (shell, model 3).

Their discretization has been shown in Figure 6. This is done to show the importance of the FEM models for optimization purposes and to contrast the results coming from different levels of detailing in the numerical model. The details available in these models are summarized in Table 3. This table firstly shows the type of elements used in the three models, their total number, and the total number of nodes. The highest number of nodes and elements are given in the first model, which is because of the FEM formulation based on the 3D elements. The third model has more than five times fewer elements and the second is a little less than the third. This is very close for the number of nodes, which are also the highest for the first model and the lowest for the second model; the highest level of detail is provided in the first model. They include the web flanges, ribs, and welds, the second model does not include welds, and the third postpones welds and ribs. This is strictly related to the number of parts and instances (provided in brackets of Table 3) created in these models—only 5 were required in the simplest third model and 563 in the first, mostly because of the very sophisticated welding required for the SIN web I-beams. This is also why the quantity of tied connections in the different models differs dramatically—only 6 for the third model and almost 1400 for the first. The latter part of Table 3. summarizes the constitutive models, analysis types, and the type and number of interactions between the modeled parts. The constitutive model applied, and the types of analyses performed for these models are the same, because of the optimization requirements. Static and static general analyses return stresses $\sigma_{ij}$, Huber–Mises stress $\sigma_{red}$, and ultimate deflection $u_{max}$.

The buckling analysis outputs the critical load ξ and the frequency analysis returns the eigenfrequency Ω. All of them are used in the checks of LS and $\beta_{g}$. The constitutive relation is set to linear with a plastic limit to conform to the standard approach of the Eurocode [39].

The topics that require further attention are the details of discretization and features included in different models. The details of discretization are shown in Figure 6, which brings us closer to the mesh used in all three studies. The first two shell models (models 2 and 3) have a quad-dominated mesh with a free meshing technique, allowing the best adaptivity of the elements to the geometry, while the volumetric model—a mixture of hexahedral and tetrahedral finite elements of both structured and unstructured meshes with different sizes. This variability is provided to optimize the time effort and computational accuracy. A structured hexahedral mesh is applied to the web and flanges, while the unstructured meshing technique is preserved in the welds; this is because of their complex geometry. The mesh of ribs and webs in the shell models is structured and composed of quad elements, while one of the flanges is a mixture of quadratic and triangular elements that adapt to the sinusoidal pattern of the web; this is visualized in Figure 6. Elements used in all computations are conventional stress-displacement-based FEs. The C3D8 is a linear brick, with eight nodes, reduced integration, and a single integration point. The C3D10 is a second-order 10-node tetrahedral element with four integration points at each tetrahedral vertex. S4R is a shell with four nodes, reduced integration, and a single central integration point. It has implemented hourglass control and finite membrane strains. S3 is a three-node triangular general-purpose shell with finite membrane strains. The simple support was modeled with linear constraints. They were placed directly below the middle of the support rib (models 1 and 2) or at the outer edge of the bottom flange (model 3) along the bottom flange width. On the left side only rotational DOF (UR1) was allowed; on the right, UR1 and displacement along the length of the girder were allowed (U2). The load was applied as the equal surface load on the entire upper flange of the girder. Its magnitude was equal to 107.14 kN/m^2^ (equivalent to 150 kN/m) including the dead load. The geometry of this girder was adopted exactly as given in Figure 5.

**Figure 7:**
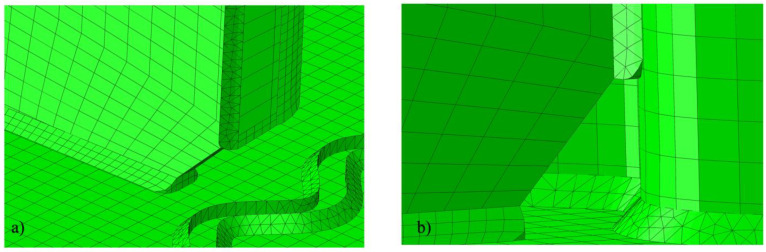
Additional views on the discretization for a volumetric model of the girder (model 1). (**a**) the details of the weld discretization; (**b**) welded connection of the ribs with the sinusoidal web.

The latter detail—features modeled—illustrates a development of numerical research. Each consecutive model made in ABAQUS brings new additional details, namely the ribs and welds depicted in Figure 7 for the first model. It is also quite important that this first model consists of three-dimensional elements. This enables not only a more accurate stress analysis through the thickness of the modeled parts but also makes possible a check of interaction between the different elements of the girder and its utilization in terms of internal stress. They both are not available in the simplified models.

### 3.2. Deterministic Limit States Analysis

Computation of the limit states is performed based on the results taken from the FEM simulations, which are summarized in Table 4, where $\sigma_{\mathrm{cr}}$*,* τ*,*${\;u}_{\max}$*,* ϖ, and ξ are shown for the vicinity of the optimal web thickness $t_{w}=56\;\mathrm{mm}$. This table firstly shows that the stresses and displacements increase together with an increase of $t_{w}$ (an increase of *W*$\;\left(t_{w}\right)$), while the ϖ and ξ increase. All these effects are desirable, because the smaller the stresses and strains, the lower the usage of the material, and, secondly, the higher the critical loads and eigenfrequencies, the higher the margin between stability loss and the current state. Moreover, an addition of the welds has rather a marginal stiffening effect, because the displacements from the second and the third model are very close, at least in the considered loading scheme. A slightly higher $u_{\max}$ for the first model comes principally from the addition of the welds and the difference between volumetric and shell FEM formulation. This is not true for the ultimate stresses and stability, both of which are significantly affected by the ribs. They decrease the ultimate stresses in the FEM for $\sigma_{\mathrm{cr}}$ and τ that are returned for the models without ribs. Interestingly, the addition of ribs directly connected to the web does not essentially change the stress flow of shear in the web, yet causes its strong reduction. This happens especially in the first model, which additionally detects quite a high shear in the entire web-flange weld, even in the middle of the girder span.

The stresses induced in the middle of the span on the outer surface of flanges are comparable in all the models, but their placement is at the flange in the shell models and in the weld of the volumetric model. This placement is also the cause for a little oscillatory effect of the maximum stresses in the first model, where small changes in the thickness must also result in a change of the mesh; this effect is not observed in the shell models. A stress state is determined in the FEM at the post-processing stage, which is the major cause of its susceptibility to all discretization changes. Such problems are not observed in displacements, being the direct results of the FEM, nor in the global characteristics of $\bar{\omega }$ and ξ; the character of stresses and their pattern is considered in a separate study.

Further, it is seen that the critical loads (*CL*) returned from the three models differ significantly for the three models. This is first because of the stiffening effect of the ribs for different global modes of buckling, and secondly because of the volumetric FEM formulation of the first model. This is evidenced in Table 4, which defines the ratio of the load at stability loss $q_{max}$ to the level of loading coming from external actions according to the Eurocode $q_{initial}$ with a subtracted initial load:

(18) $$\frac{q_{\max}}{q_{initial}}=CL+1.$$

*CL* is much lower for the first model than for the others. It is because it was the only one that returned a local buckling of the support rib. Other models returned only the global losses of stability, the first of which was always rotation-torsional. They occur for quite a high magnitude of the *CL* (critical load ratio of over 4.45 or 7.37) for a girder, which is initially loaded to 88.4% of its ultimate bending capacity according to the assumptions of the Eurocode. The critical load in the volumetric model is much lower, but all the critical modes of behavior until the 25th one can be easily avoided by an increase in the thickness of the support or, preferably, a change in its geometry from plate to corrugated (insensitive to local buckling). Quite interestingly, the addition of the ribs increased the CL by about 50% for the shell model. No buckling is detected in the web itself, either by stiffened or unstiffened models. This proves the high contribution of the wavy web to the overall stability of the girder and the reasonable significance of its thickness, whose increase enlarges the critical load. This increase is consistent with an engineering intuition, but it does not stop the underneath mechanism leading to the buckling of the girder. Contrary to the results of the critical load, these for eigenfrequency show almost perfect agreement for models 1–2 and give a slightly lower quantity for the third one. This difference is increasing for the higher eigenfrequency modes (see Table 4), which is caused by the change in the linear dead load of a girder due to the stiffening ribs and welds, yet does not significantly hinder the character of these modes.

It must be additionally mentioned that a choice of the proper ultimate stress or displacement for purposes of LS and reliability restrictions is not a trivial task, especially when a complex 3D structure is considered and modeled with the use of shell or volumetric FEM elements. The global ultimate stress does not necessarily give a proper condition for the ULS and may turn out to be inappropriate for restrictions. Even for such conceptually uncomplicated structural elements as a simply supported beam, the ultimate stress may lie in other places than those distinguished by the beam theory (as happens in this study). In such conditions, reliability shall be checked not only for the location of the ultimate stress but also for these other locations detected in the beam theory, because the material in these places may be susceptible to local instability (as in all plate structures). Because of this, the ultimate stress allowed in such locations may be highly reduced and conversely, the material can be locally stronger in a location of ultimate stress predicted by the FEM (e.g., when some confinement exists). Therefore, in all complex structures, one should always first determine its possible weak points and check the reliability for all of these, not only the one for the ultimate stress or displacement revealed by the FEM results.

### 3.3. Probabilistic Aspects

The determination of the reliability indices $\beta_{g}$ and related optimization procedures have been both programmed and completed in the computer algebra system MAPLE. The WLSM is based on the discrete results of three FEM models (see Table 3) and for three types of weights, Dirac, equal, and triangular (see Table 2). The input random parameter is web thickness with a mean value taken from the objective function W and updated in each loop of the optimization. The probabilistic density function (PDF) of this thickness is Gaussian and we treat here two separate random problems. The first is at t=0 and is connected with a fabrication error (manufacturing imperfection) of this thickness with coefficient of variation (COV) in the range of *α*$\left(\omega_{0}\right)\in \;\left\{0;0.15\right\}$, and a second, where in addition to $\omega_{0}$, a second random variable is corrosion penetration depth, where time t is a design variable in a range of *t*∈〈0;50〉 years. This second check is also a final and most severe restriction in the optimization loop because it takes into consideration the degradation of the girder with time. The formulation of the probabilistic moments and coefficients is here threefold:

- First of all, the coefficients are computed by direct differentiation of the random variable computed from the response function together with its PDF; this is called the semi-analytical approach (AM);
- Secondly, the generalized iterative stochastic perturbation technique is applied with up to 10th order approximation of the response function by the Taylor expansion—including the first 10 terms of this expansion; this is called the SPT [42];
- Finally, the crude Monte Carlo simulation (MCS) is used to return these coefficients; this is called the MCS.

Such an approach is selected for purposes of comparison between these three methods and also for redundancy so that even if one method fails in a specific optimization step or for the specific g, the indices could still be calculated and compared. A crude MCS with $5\cdot 10^{5}$ trials is chosen to dissolve all the doubts about the accuracy of a more refined Monte Carlo method. The spectrum of the web thickness used in each step is $t_{w,\;i}\pm 5\;\mathrm{mm}$ with a difference of 1 mm for each computation, so that the response function is optimized based on 11 FEM results around the mean value defined at each optimization loop. The algorithm developed in MAPLE fully encompasses the process of reliability indices β (see Figure 1 and Figure 3) and $\beta_{\sigma_{\mathrm{cr}}},$ and for *t*∈ 〈0;50〉 only $\beta_{u_{\max}}$ is restricted, which is done for the computation efficiency and simplicity of this example.

### 3.4. Reliability Restrictions $\beta_{g}$

The final reliability calculations are shown for the optimized W, for which $t_{w}=56\;\mathrm{mm}$. The structure of the reliability assessment for this W is divided into three main sections:

- The first of these determines the influence of different types of weighting schemes and FORM vs. SORM formulations. It takes the manufacturing imperfection as an input variable, is based on the volumetric FEM model, and is calculated for t=0.
- The second analysis uses a Dirac-type of weighting scheme and compares the results of stochastic equations with manufacturing imperfection as a random parameter for all three FEM models and deterministic results. This is done to highlight the impact of the FEM and model accuracy on the girder’s output reliability. It is also performed at t=0.
- The last study incorporates two random variables, including the fabrication imperfection $\omega_{0}$ and corrosion penetration depth ω, and serves as the last restriction for the optimization purposes of this girder.

This is done firstly to show the results for the optimized W and secondly to emphasize the most important factors in the optimization, i.e., the FEM model, order of reliability assessment, and type of the weighting scheme in the optimization of the WLSM. Three independent methods of computation, AM, MCS, and SPT are used for simultaneous verification of the results. The probabilistic moments and coefficients are shown only for one, most restrictive limit function and solely relative to the weighting scheme, which is done to show their general outlook and present relation with an input uncertainty. The other ones return analogous results.

#### 3.4.1. Initial Restriction of $\beta_{g}$—WLSM Weighting Scheme

**Figure 8:**
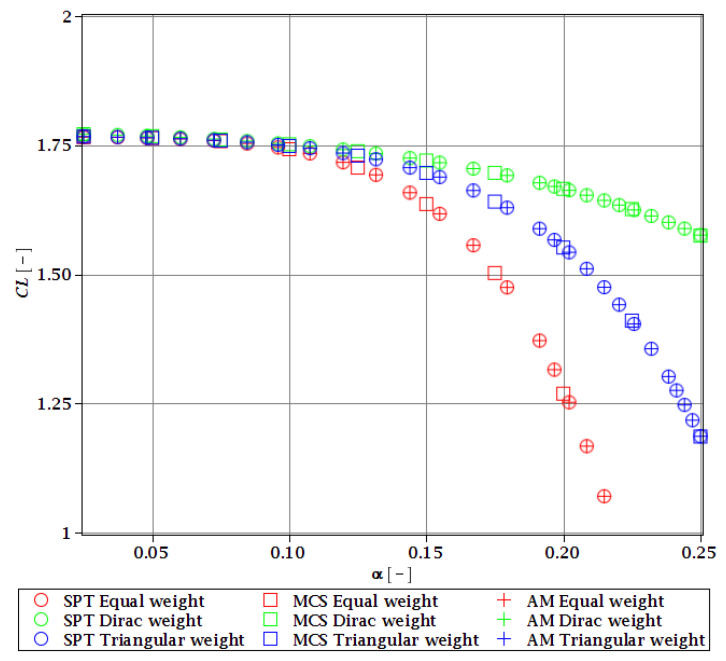
Expectations of the critical load for different types of weights.

**Figure 9:**
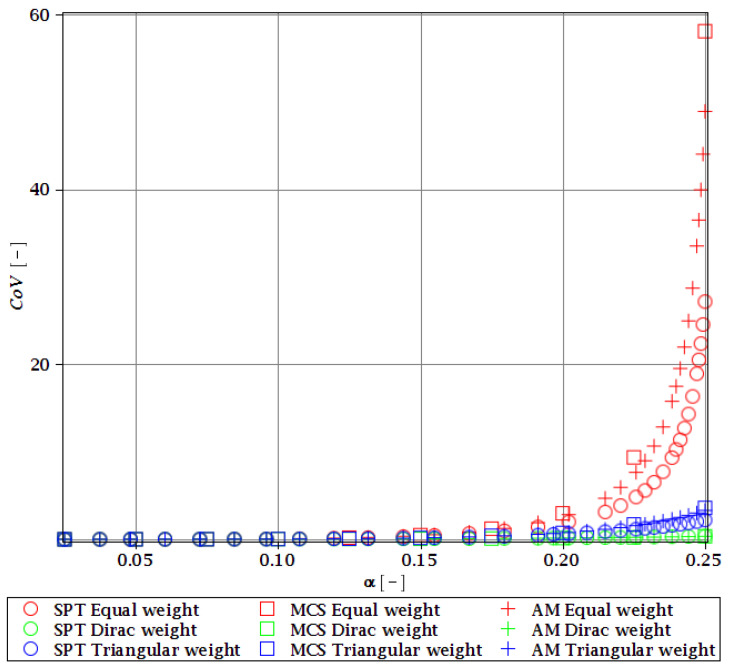
CoV of the critical load for different types of weights.

**Figure 10:**
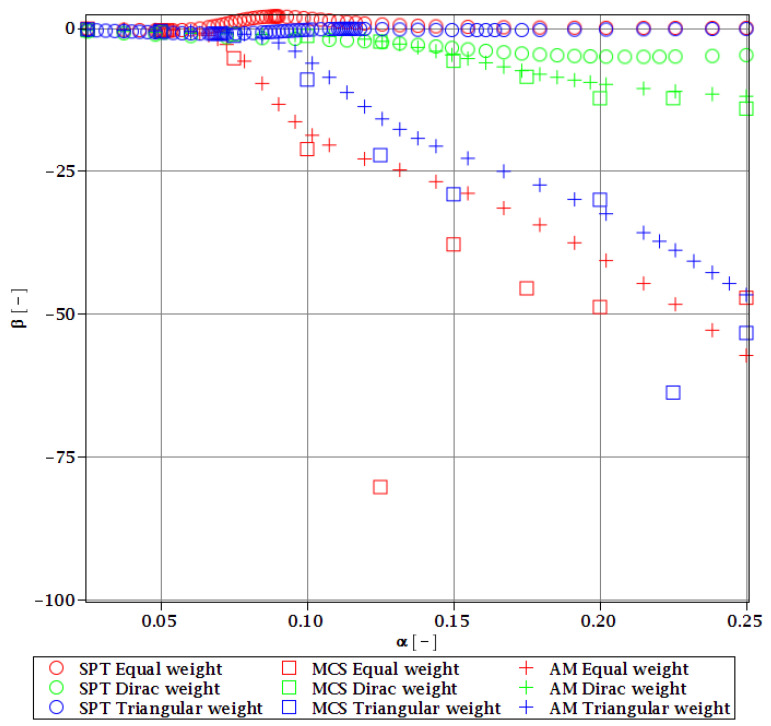
The skewness of the critical load for different types of weights.

**Figure 11:**
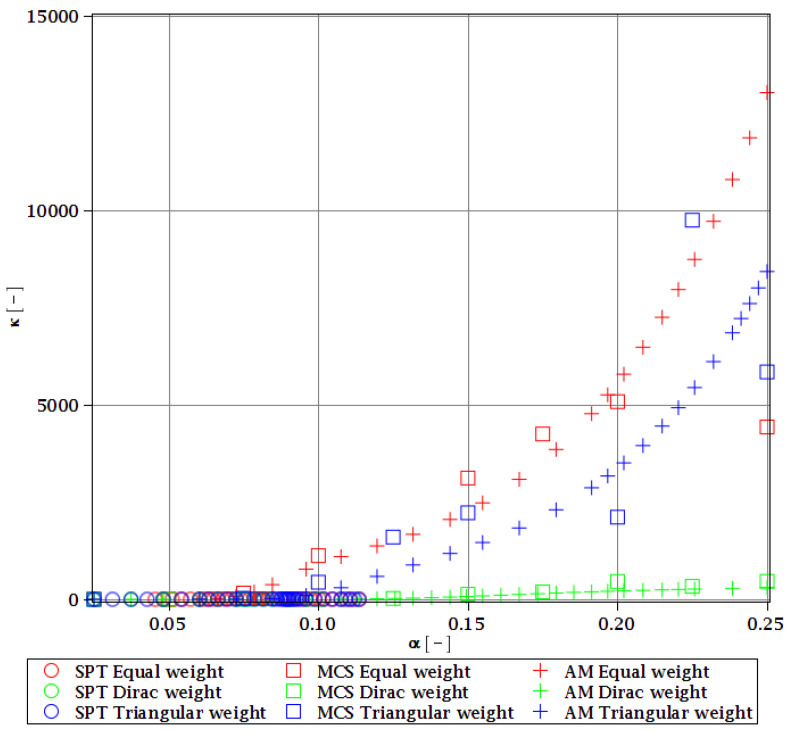
Kurtosis of the critical load for different types of weights.

**Figure 12:**
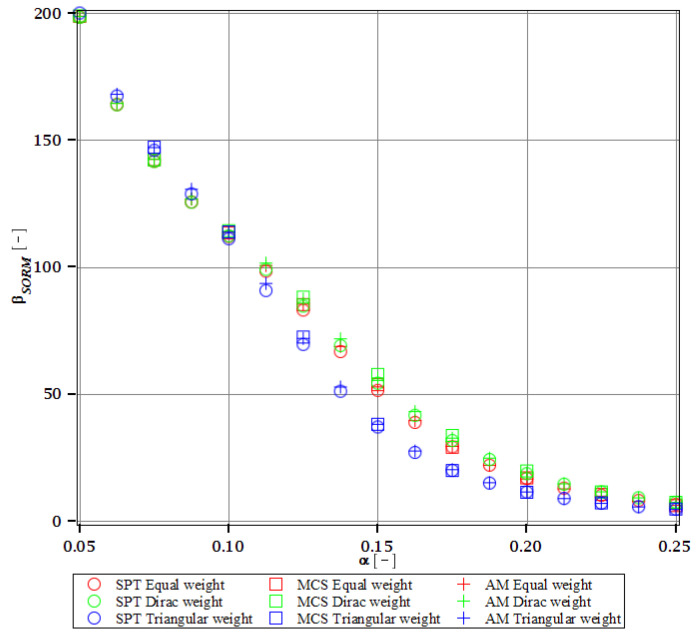
Reliability index of the corrugated I-beam for different types of weights according to the second-order reliability method (SORM)—displacement.

**Figure 13:**
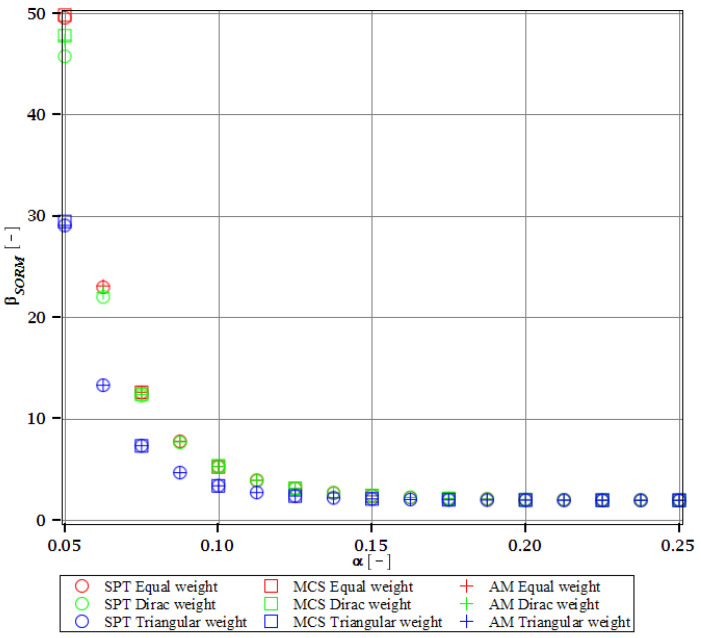
Reliability index of the corrugated I-beam for different types of weights according to the second-order reliability method (SORM)—normal stress.

**Figure 14:**
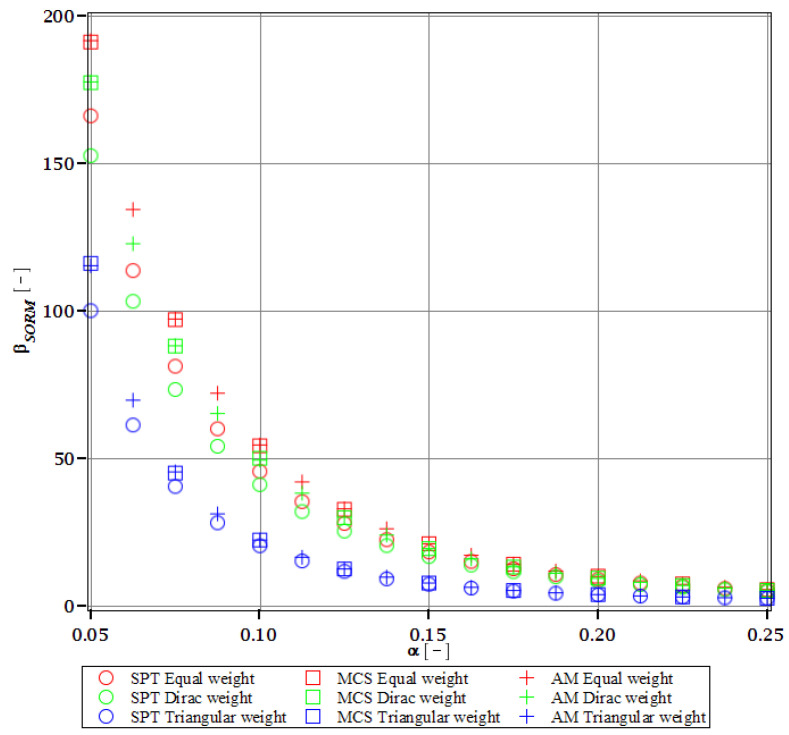
Reliability index of the corrugated I-beam for different types of weights according to the second-order reliability method (SORM)—shear.

**Figure 15:**
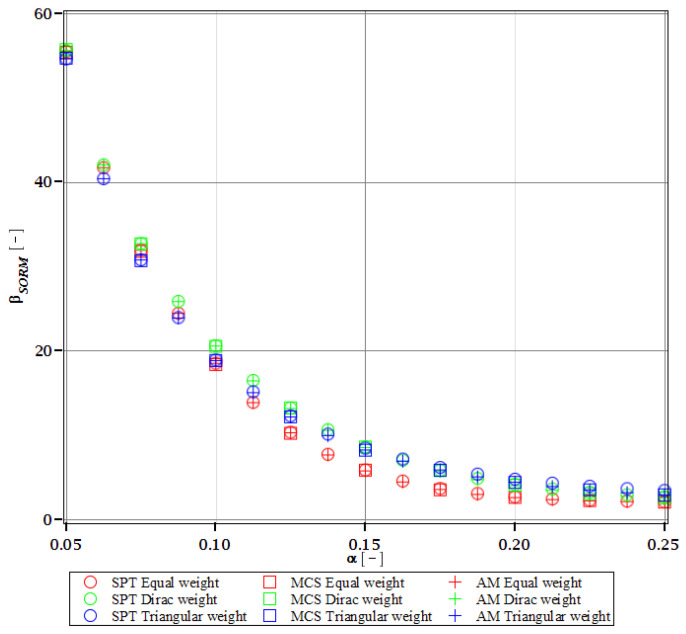
Reliability index of the corrugated I-beam for different types of weights according to the second-order reliability method (SORM)—eigenfrequency.

**Figure 16:**
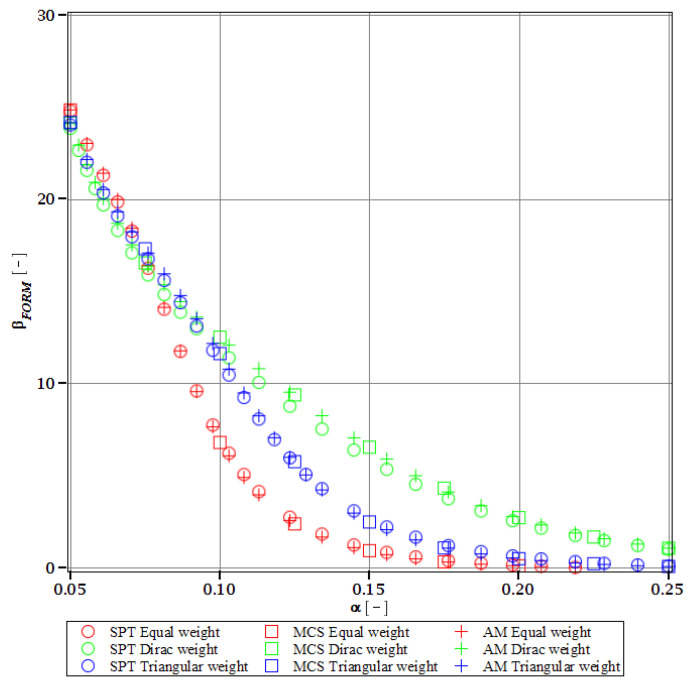
Reliability index of the corrugated I-beam for different types of weights according to the first-order reliability method (FORM)—critical load.

**Figure 17:**
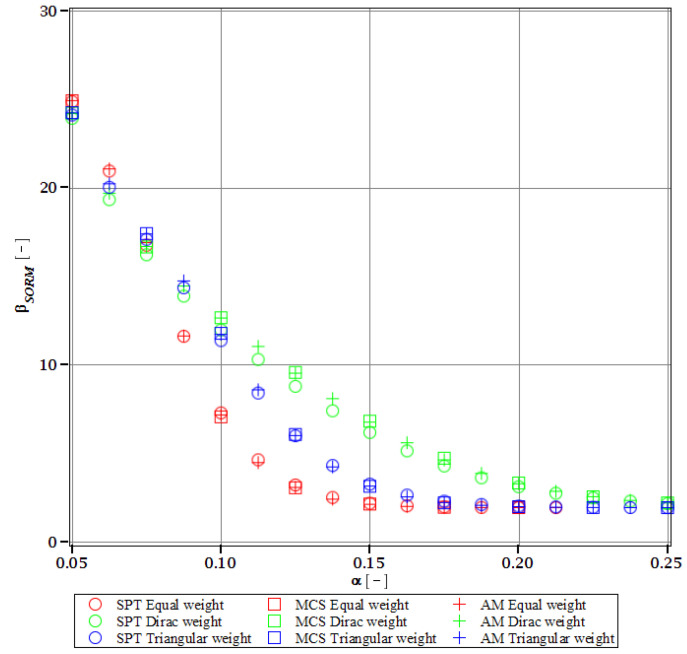
Reliability index of the corrugated I-beam for different types of weights according to the second-order reliability method (SORM)—critical load.

Computations of reliability-induced restrictions for the girder at t=0 include the determination of the first four probabilistic moments and coefficients of all the limit functions considered, i.e., $E_{g}\left(\alpha \left(\omega_{0}\right)\right),$ $\alpha_{g}\left(\alpha \left(\omega_{0}\right)\right),$ $\beta_{g}\left(\alpha \left(\omega_{0}\right)\right)$, and $\kappa_{g}\left(\alpha \left(\omega_{0}\right)\right),$ and its reliability index $\beta_{g}\left(\alpha \left(\omega_{0}\right)\right)$. They are depicted in Figure 8, Figure 9, Figure 10, Figure 11, Figure 12, Figure 13, Figure 14, Figure 15, Figure 16 and Figure 17 and are computed relative to the coefficient of variation of web thickness inflicted by an uncertain fabrication error *α*$\;\left(\omega_{0}\right)$. This ensures an easy way of check of these restrictions for the chosen level of uncertainty and avoids the need for repetition of the entire optimization process in the case when its level is slightly increased. The full results are shown only for the most restrictive condition, which is a critical load (CL). The results of all other ones are limited solely to the index of reliability according to SORM; this is done for the brevity of the results.

The lower probabilistic coefficients for the limit state based on stability criterion $E_{\xi }\left(\alpha \left(\omega_{0}\right)\right)$ and $\alpha_{\xi }\left(\alpha \left(\omega_{0}\right)\right)$ firstly show that they both are highly affected by the uncertainty caused by the manufacturing error. The expected values (Figure 8) decrease, and the coefficient of variation (Figure 9) increases together with an increase in this uncertainty and the rate of this change always increases. Interestingly, the changes in the $E_{\xi }$ are up to 50% and the coefficient of variation is multiple times higher than for the input. The three methods of computation show a perfect agreement.

The skewness $\beta_{\xi }\left(\alpha \left(\omega_{0}\right)\right)$ and kurtosis $\kappa_{\xi }\left(\alpha \left(\omega_{0}\right)\right)$ for the critical load depicted in Figure 10 and Figure 11 represent a converse character. The skewness is predominantly negative, while the kurtosis is positive. They both have quite a strong relationship with the input uncertainty, and they reach very high magnitudes of up to 120 for $\beta_{\xi }$ and 13,000 for $\kappa_{\xi }$. A scatter of the three stochastic methods is quite high, but still, the AM and MCS demonstrate a quite comparable trend. The SPT is effective here only up to α=0.1, but it is the fastest.

The indices of reliability $\beta_{\sigma }$, $\beta_{\sigma_{\mathrm{red}}}$, $\beta_{\tau }$, $\beta_{u_{\max}}$, $\beta_{\Omega }$, and $\beta_{\xi }$ include all the relevant limit states and are all shown in Figure 12, Figure 13, Figure 14, Figure 15, Figure 16 and Figure 17. They principally show a high converse dependence on the input uncertainty of manufacturing error and are also affected by the WLSM weighting scheme. The lowest initial reliability is reported for the critical load (see Figure 16 and Figure 17). This is a direct effect of two main factors. The first of these is the fact that even the deterministic safety is quite small here (function close to one, which constitutes its lower limit, see Table 4). The second is connected with a magnitude of the *CoV*, which is very high for this state function (Figure 9) and has a strong exponential character relative to the input *CoV*. With an increasing input uncertainty, this smallest index rapidly decreases, but still from approx. *α*$\;\left(\omega_{0}\right)=0.07$ the lower bound of reliability is governed by the normal stress shown in Figure 13. The indexes also depend upon the type of weight of WLSM. This difference is the strongest for the two indices constituting the lower bound of the reliability, namely the normal stress and the critical load, and additionally for the shear (see Figure 13, Figure 14 and Figure 16). This is because their first two probabilistic moments show the highest dependence on the type of weight applied.

The character of a relationship between the reliability index and an input uncertainty is not always smooth and without local inflection points. The indices for the ultimate displacement, as well as critical load, show small fluctuations through their course, especially around *α*$\;\left(\omega_{0}\right)=0.10$. Such fluctuations are intriguing, but for now, their cause has not been determined.

The last comparison is the FORM vs. SORM index of reliability, which is given based on critical load, the most difficult reliability restriction at t=0. It is based on Figure 16 and Figure 17.

The graphs according to both orders have a very similar character. This is expected, because the applied SORM is based on the Gaussian input probability density and, therefore, these indices should be comparable. An important observation is that $\beta_{g}$ according to SORM is less dependent on the type of weight and has a little higher magnitude for an extensive input uncertainty. Finally, the SORM approach corrects the errors coming from the FORM, i.e., the diverging or scattered character of $\beta_{g}$ for some limit functions. An additional observation is concerned with the interchangeability of these three probabilistic methods applied in restrictions of the reliability index; all the methods show an almost perfect agreement for all the state functions. Due to this, when only one stochastic variable is taken into consideration, all the methods can be used alternatively. In such conditions, the most preferable one seems to be the stochastic perturbation method (SPT), which is not dependent on a direct derivation, does not require a considerable number of trials (as the MCS), and is also the swiftest.

The last, yet most important, observation is a total limit of input uncertainty coming from the fabrication imperfection that allows for fulfilling the restriction of reliability $\beta_{g}$. This limit differs for all the state parameters and ranges from *α*$\;\left(\omega_{0}\right)=0.09$ to around *α*$\;\left(\omega_{0}\right)=0.21$. The lowest one constitutes a total limit, and therefore the objective function with optimum $t_{w}=56\;\mathrm{mm}$ is *α*$\;\left(\omega_{0}\right)=0.09$. This limit corresponds to the limit function of critical load (ξ). This is a reasonable result because for most of the constructional elements the fabrication error causes uncertainty in $t_{w}$ lower than $\left(\omega_{0}\right)=0.05$. Nonetheless, reliability restrictions at t=0 are not the most rigorous, which undoubtedly are the ones connected with a joint effect of corrosion and the considered fabrication error. Improving the resistance of the girder to stability loss would be enough to increase the overall reliability of this girder at *t* = 0, but it may not be sufficient to improve reliability in the corrosive environment.

#### 3.4.2. Initial Restriction of $\beta_{g}$

**Figure 18:**
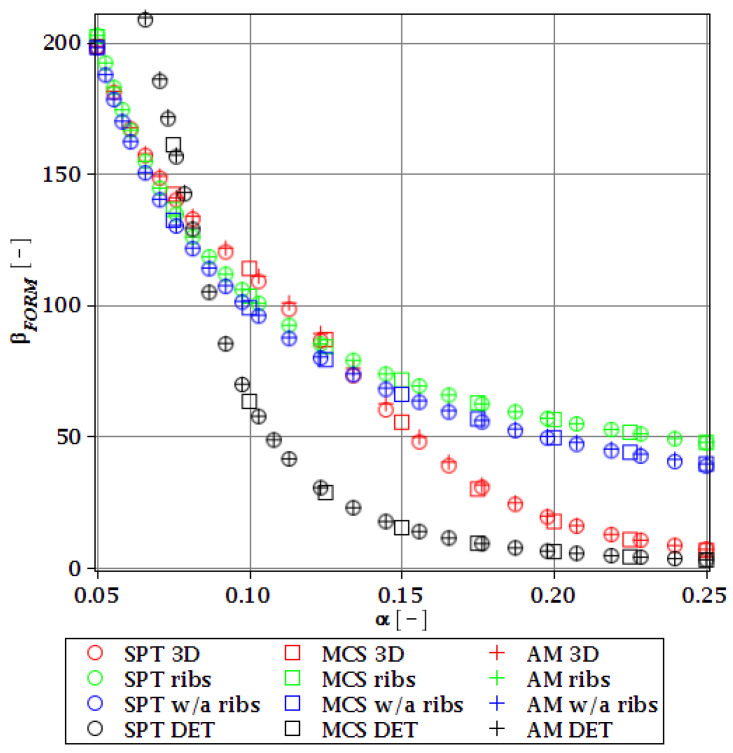
Reliability index of the corrugated I-beam for three ABAQUS models according to FORM and based on the deflection.

**Figure 19:**
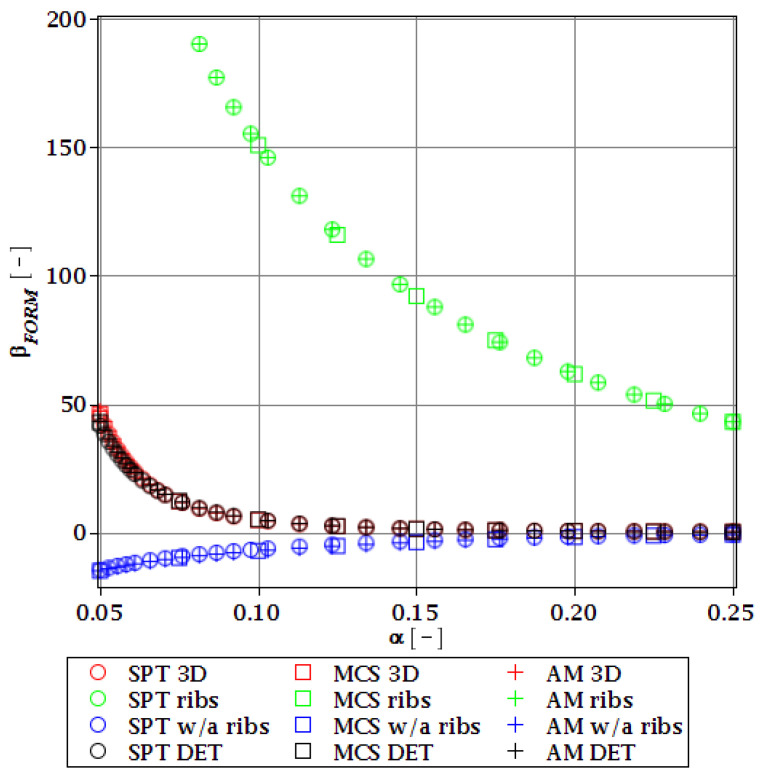
Reliability index of the corrugated I-beam for three ABAQUS models according to FORM and based on the ultimate normal stress.

**Figure 20:**
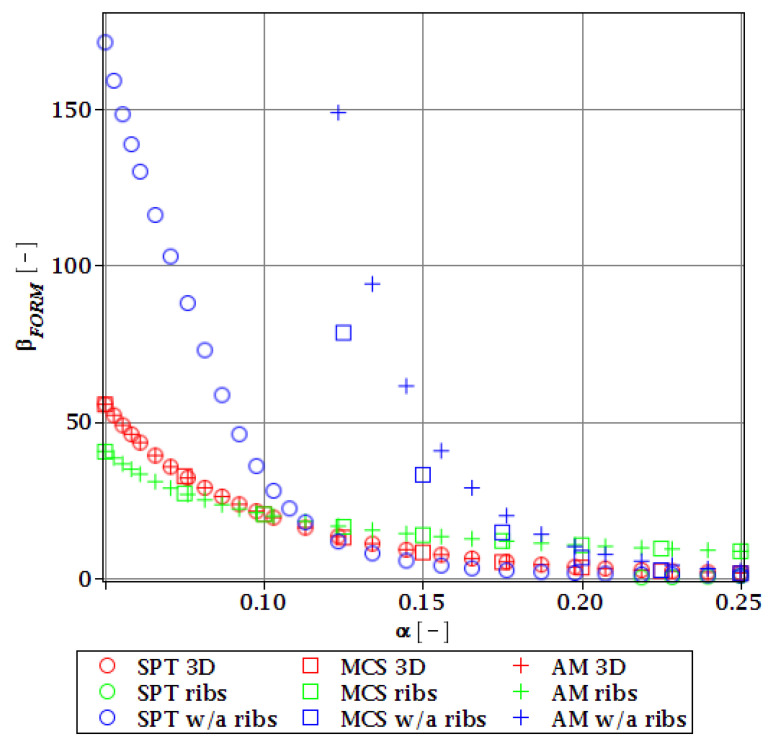
Reliability index of the corrugated I-beam for three ABAQUS models according to FORM and based on the eigenfrequencies.

**Figure 21:**
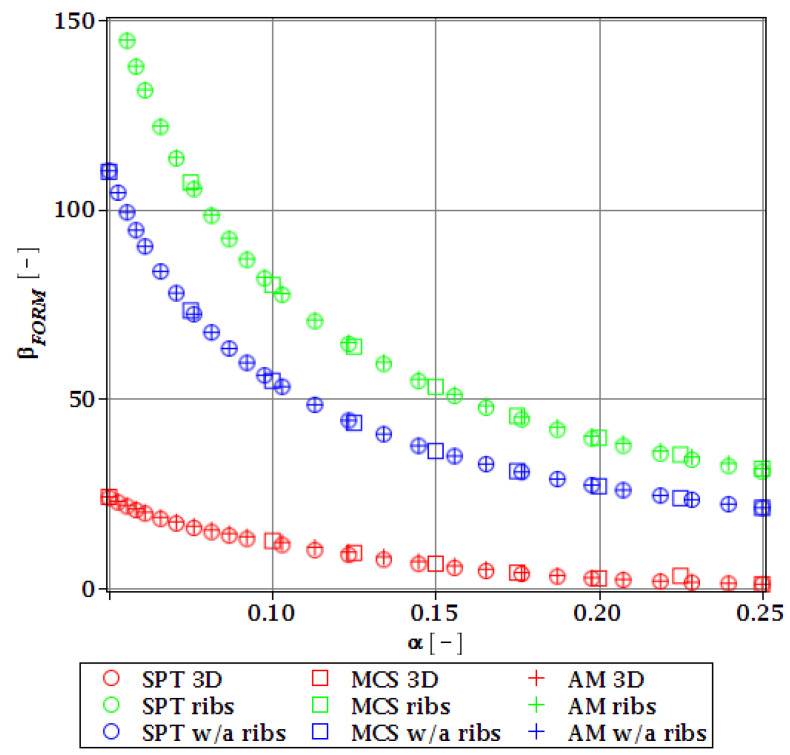
Reliability index of the corrugated I-beam for three ABAQUS models according to FORM and based on the critical load.

The differences in initial reliability restriction fulfillment for the three models are shown in Figure 18, Figure 19, Figure 20 and Figure 21. They depict the FORM indices for the four most important limit states, i.e., $\beta_{\sigma }$, $\beta_{u_{\max}}$, $\beta_{\Omega }$, and $\beta_{\xi }$ in the function of the *α*$\;\left(\omega_{0}\right)$ and for t=0. These plots are principally presented to highlight the importance of the choice of the FEM model type and its accuracy in fulfillment of the reliability restrictions for the objective function. They are all computed with the use of the Dirac weighting scheme and with three alternative probabilistic methods, MCS, AM, and SPT. These indices perfectly justify the purpose, being vastly dependent on the model type and almost uniform for all the probabilistic methods. Therefore, it is highly recommended to put more effort during the modeling process of the FEM and into the collection of data coming from these simulations than into the choice and scrutiny of the probabilistic method. The probabilistic coefficients bring no more information for optimization purposes and therefore they are not included. One may refer to Figure 8, Figure 9, Figure 10 and Figure 11 for the required information.

From Figure 18, Figure 19, Figure 20 and Figure 21 it is first seen that the reliability margin decreases together with an increase of *α*$\;\left(\omega_{0}\right)$. It is in the range of 50 ÷ 150 for a small *α*$\;\left(\omega_{0}\right)\;<\;0.01$, but it rapidly decreases and reaches zero for *α*$\;\left(\omega_{0}\right)\;\in \left(0.10;0.14\right)$; this is still much higher than typical uncertainty caused by the fabrication imperfection. The second observation concerns the inexistence of a limit state setting the reliability restriction for the entire *α*$\;\left(\omega_{0}\right)$. For each level of uncertainty, this limit is set in different LS, and therefore the topological optimization is not trivial—each limit state depends on multiple parameters of the model. The highest restriction is decisive, which is not unique for the different models. On the other hand, the first model is also the most restrictive for a majority of the cases and because it is also the most detailed one, it should be the one taken for the final optimization. This is because it had the smallest margin in the LS. An alternative approach is the inclusion of results from all the models for optimization purposes, but this would cause almost threefold longer computations because the FEM solution is very computationally-intensive. The correspondence between the three methods is almost perfect for all the four considered reliability indices and for all the FEM models, with only one exception of Figure 20 for the third, most simplified model (shell without ribs), for which neither of the methods converges.

One of the observations which should undoubtedly be highlighted is the fact that for all the limit states the reliability indices according to different models diverge not only in their initial value but also in the strength of their relation to *α*$\;\left(\omega_{0}\right)$. A very good example is an index based on the deflection (Figure 18), which starts very high for the deterministic model and just over *α*$\;\left(\omega_{0}\right)$ = 0.07 crosses all the other indices to become the lowest (to constitute a lower limit) for all higher *α*$\;\left(\omega_{0}\right)$. One more interesting relation is unveiled by the reliability index based on the ultimate normal stress (Figure 19) and computed according to the shell model (third model) which shows a negative value of the index. This is a direct outcome of an existence of the unacceptably high stress exceeding the plastic limit of the construction steel already in the FEM results. The existence of the negative index is incorrect and should by all means be excluded from further analysis. Nevertheless, unlike in *α*$\;\left(\omega_{0}\right)$, its negative value gives a piece of important information for optimization purposes—that the element is not fulfilling restrictions already in the process of the limit states, and before reliability checks, a new optimization loop with a new value of W should have been already started.

#### 3.4.3. Durability Analysis with $\beta_{g}$

**Figure 22:**
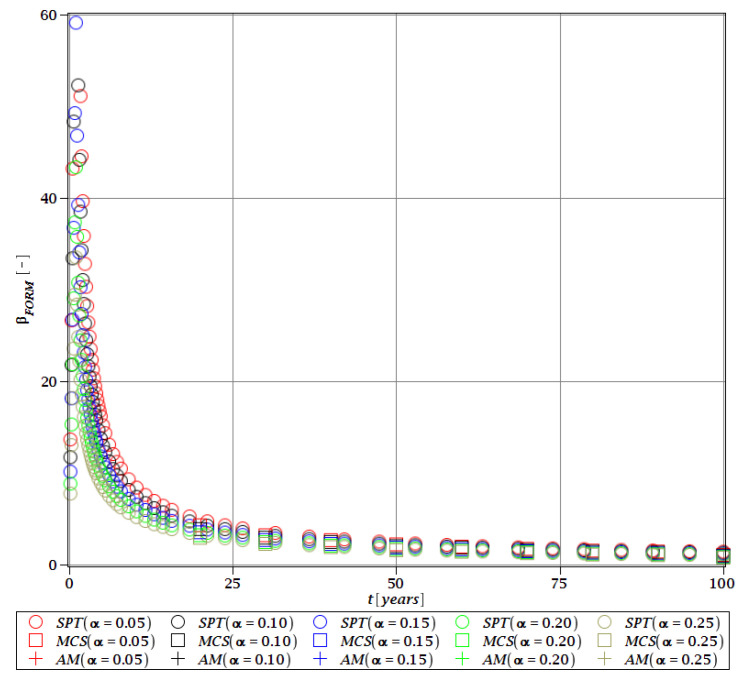
Reliability index of the corrugated I-beam for different types of weights according to FORM—deflection (SLS), the second-order polynomial.

The last and final restriction of the optimization problem is the reliable service life of the constructional element, which is set as t=50 years. This restriction is formulated with the use of the FROM index of $\beta_{g}$ and presented for the limit state of $u_{\max}.$ It is computed for the joint impact of the corrosion penetration depth and the manufacturing imperfection, both being random and uncorrelated. Reliability is calculated with three probabilistic methods, i.e., AM, MCS, and SPT. Corrosion is modeled according to the third model from Table 1 with two random parameters A and B, while the uncertainty of initial imperfection is set within the following bounds of *α*$\;\left(\omega_{0}\right)\in \;\left[0.05;0.25\right]$ with a Gaussian PDF. This imperfection is introduced during the production of the beam and is considered time-independent, while the corrosion process is described by a time series. The response function utilized for probabilistic calculations is calculated with an inner optimization problem and WLSM is based on the Dirac weighting scheme. The result is presented for the optimized objective function *W*$\left(t_{w}=56\;\mathrm{mm}\right)$ and based on the most refined first FEM model. This final reliability restriction is depicted in Figure 22 as a function of service time *t* ∈ (0;50) years. It firstly shows that the limit of 1.8 is reached at around 60 years, and the margin of the restriction is within the stop criterion defined for this example as $\beta_{T}=0.3$. Secondly, the uncertainty of an initial manufacturing imperfection only marginally affects the final result, and this is why its sole impact was checked in the preceding step of the optimization loop. This index starts at$\beta_{g}=60$, sharply decreases in a convex manner with a decreasing slope and has an apparent limit of $\beta_{g}≅\;0$. This index shows a very good coincidence of the two probabilistic methods, the SPT and MCS. The third, semi-analytical method (AM) diverges from the others, and this is why it is not reported in Figure 22. This exemplifies the usability of this triple redundant method, which allows a successful check of restriction even when one of the probabilistic methods turns out to be unavailable or divergent for a specific step.

## 4. Concluding Remarks

This paper presents an optimization framework for topological problems in the domain of civil engineering. It is exemplified by a successfully optimized simply supported SIN web I-girder. The main novelty in this paper is the concept of reliable service life prediction and its application to a computationally demanding structure. It allows automation of the reliability-based design of custom structural elements. The principal objective of the proposed RBDO algorithm is a determination of the best topology that satisfies all the design restrictions applicable to civil engineering structures during their service life. Restrictions include the limit states, i.e., the ultimate limit state and serviceability limit state, stability, and vibrations in the deterministic and reliability context. The limit functions are applied directly after the FEM results. The optimization loop consists of subsequent verification of physical and geometrical restrictions, FEM problem solution, verification of all deterministic design restrictions, and finally verification of reliability-based restrictions. This is done for a specified service life of construction. It could be applied to a wide variety of structural elements and entire structures; the limit functions could be obtained analytically, by BEM, FEM, neural networks, or with the use of any other algorithm that outputs the required state parameters.

The most critical points of this algorithm include the calculation of the representative limit functions for local state parameters, such as stresses and strains, FEM detailing, and interpretation of FEM results. For this reason, three concurrent models were proposed and shortly contrasted in the optimized solution; in current maturity, the algorithm uses a deterministic non-gradient search. It is intended for replacement in future works by a more effective method, such as neural networks.

The proposed algorithm is applied to a practical example of the SIN web I-girder. This provides additional insight into the reliability of such elements, as well as their susceptibility to loss of stability, vibrations, and deformability. It also adds some valuable remarks to the FEM modeling in RBDO problems. The results obtained in this work confirm that numerical modeling precision significantly affects the optimization outcome. This is because its choice has a direct effect on the stress state of the girder and its buckling. Different FEM models applied in this study return important dissimilarities in stress distribution, its maximum values, and placement of peaks. This is also true for buckling loads and their patterns. It is also exemplified here that the choice of the FEM formulation (volumetric vs. shell), as well as the finite element type and order, significantly affects the optimization. Furthermore, it is difficult to set the required level of detail in the FEM model and the minimum amount of FEs before the RBDO. This is because the buckling load together with ultimate stresses and deflections have no clear correlation to the model accuracy. Therefore, a decrease in the level of geometric and computational precision may hide significant design problems. They include low buckling mode or high-stress peaks in the welds that are omitted by simplified models and lead to an overestimation of the overall reliability of this structure. Importantly, only the most detailed model revealed additional instabilities in the girder occurring at much smaller loads than the others and having a local, rather than global character.

The corrosion process affected the considered example in terms of both reliability and structural capacity. Its evolution increased the internal stresses and decreased critical loads. The influence of the initial fabrication error on the service life of the exemplary structure was marginal and may be omitted in future research. On the contrary, steel and environment type had a much more substantial effect on the service life of an exemplary structure. This is because of its direct effect on the evolution of corrosion depth and its uncertainty. Please note that maintenance, such as painting or plating, is not taken into consideration in the current algorithm. Performed regularly, it will increase the reliable life of the structure. Its inclusion is planned in future research.

The triple probabilistic calculations applied in this study provided a piece of important information on the convergence of probabilistic calculations that would be otherwise unavailable. On the other hand, their mutual application slowed down the optimization process. Computational time reduction could be achieved by limiting the RBDO to a single probabilistic method. The ISFEM is the most recommended in this case. It is the fastest and significantly accelerates the optimization process. Its accuracy is a little lower than that of the MCS, but the difference proved to be marginal for the considered example.

## Figures and Tables

**Table 1:** Statistical parameters of corrosion for various steel types [43].

Parameters	Carbon Steel		Weathering Steel	
	A (10^−3^ mm)	B	A (10^−3^ mm)	B
Rural environment				
Expectation	34.0 (model 1)	0.65	33.3 (model 2)	0.498
CoV	0.009	0.10	0.34	0.09
Urban environment				
Expectation	80.2 (model 3)	0.539	50.7 (model 4)	0.567
CoV	0.42	0.40	0.30	0.37
Marine environment				
Expectation	70.6 (model 5)	0.789	40.2 (model 6)	0.557
CoV	0.66	0.49	0.22	0.10

**Table 2:** Selected WLSM polynomial approximations of the extreme displacement versus random input.

Displacement WLSM Polynomial Choice for Different Weights										
No.	$P_{o}$	$C_{WLSM}$			$E_{WLSM}$ [10^−2^]			$\alpha_{WLSM}$ [10^−5^]		
		$W_{SD}$	$W_{ST}$	$W_{SE}$	$W_{SD}$	$W_{ST}$	$W_{SE}$	$W_{SD}$	$W_{ST}$	$W_{SE}$
1	1st, f	0.994	0.9941	0.9941	2.105	2.166	2.124	6.216	6.414	6.554
2	3rd, f	0.9959	0.9959	0.9958	1.722	**1.720**	1.770	4.282	4.293	4.333
3	5th, f	0.9962	0.4008	0.0016	1.65	50.82	123.6	3.902	3063	18.530
4	6th, f	0.9966	0.0954	0.1556	**1.401**	202.1	58.01	3.492	66.200	446.5
5	9th, f	**0.9969**	0.3295	0.3789	1.427	67.29	57.93	**3.207**	6014	421.3
6	10th, f	0.9954	0.1112	0.3769	1.752	587.1	53.35	4.762	42.935	336.6
7	6th, p5	0.996	0.9958	0.9952	1.754	1.82	1.794	4.184	4.311	4.521
8	11th, p5	0.996	**0.996**	0.9958	1.748	1.739	1.758	4.111	**4.113**	4.388
9	15th, p5	0.9958	0.9958	0.9957	1.747	1.751	1.764	4.324	4.337	4.414
10	9th, p4	0.9958	0.9958	0.9958	1.739	1.743	**1.758**	4.319	4.333	4.389

**Table 3:** Details of the full-scale FEM models.

Model No.	Elements			Number of Parts in the Model	Details Modeled	Constitutive Model	Types of Analysis	Interaction Type, Quantity
	Type	Total Number of FEs	Total Number of Nodes					
1.	C3D8R	573,043	925,741	93 (563)	- Web - Flanges - Ribs - welds	Linear elastic with the plastic limit	- Static - Static, general - Buckling - Frequency	Tie, 1392 connections
	C3D10	152,460						
2.	S4R	77,422	85,777	10 (55)	- Web - Flanges - Ribs			Tie, 178 connections
	S3	1768						
3.	S4R	100,800	221,200	5 (5)	- Web - Flanges			Tie, 6 connections

**Table 4:** Comparison of the results from the three FEM models.

*t_w_*	$\sigma_{cr}\;[\mathrm{MPa}]$			τ [MPa]			$u_{max}\;[\mathrm{cm}]$			ϖ [Hz]			ξ (CL)		
	Model No.			Model No.			Model No.			Model No.			Model No.		
	3	2	1	3	2	1	3	2	1	3	2	1	3	2	1
51	484.8	212.0	225.6	127.6	67.45	87.12	7.126	7.206	7.710	1.39	1.37	1.73	4.398	7.304	1.708
52	484.5	211.7	229.	127.2	67.0	89.95	7.117	7.198	7.702	1.39	1.37	1.73	4.409	7.317	1.716
53	484.2	211.4	232.7	126.9	66.69	92.92	7.108	7.191	7.698	1.4	1.36	1.72	4.420	7.329	1.725
54	483.9	211.1	234.2	126.5	66.35	81.78	7.100	7.183	7.686	1.4	1.36	1.72	4.431	7.342	1.737
55	483.7	210.8	236.0	126.1	66.02	82.00	7.091	7.175	7.681	1.4	1.36	1.72	4.442	7.356	1.753
56	483.3	210.6	234.5	125.7	65.70	83.51	7,084	7.168	7.673	1.4	1.36	1.71	4.453	7.369	1.778
57	482.4	210.3	231.6	125.2	65.39	84.06	7.076	7.161	7.669	1.4	1.35	1.71	4.464	7.383	1.776
58	481.7	210.0	235.4	124,8	65.10	84.05	7.069	7.153	7.656	1.39	1.35	1.71	4.475	7.399	1.784
59	481.0	209.7	227.6	124.4	64.78	85.71	7.061	7.145	7.653	1.39	1.35	1.71	4.487	7.414	1.802
60	480.3	209.5	238.9	123.9	64.51	85.22	7.054	7.138	7.651	1.39	1.34	1.70	4.498	7.430	1.810
61	479.6	209.2	229.0	123.5	64.24	84.57	7.048	7.131	7.642	1.39	1.34	1.70	4.510	7.445	1.812

## Data Availability

Not applicable.

## References

[1] Sophianopoulos D.S., Deri A.E. (2017). Steel beam–to-column RBS connections with European profiles: I. Static optimization. J. Constr. Steel Res..

[2] Sigmund O. (1997). On the design of compliant mechanisms using topology optimization. Mech. Based Des. Struct. Mach..

[3] Lewiński T., Czarnecki S., Dzierżanowski G., Sokół T. (2013). Topology optimization in structural mechanics. Bull. Pol. Acad. Sci.-Tech..

[4] Lopez C., Baldomir A., Hernandez S. (2018). The relevance of reliability-based topology optimization in early design stages of aircraft structures. Struct. Multidisc. Optim..

[5] Frangopol D.M., Maute K. (2003). Life-cycle reliability-based optimization of civil and aerospace structures. Comput. Struct..

[6] Schuëller G.I., Jensen H.A. (2008). Computational methods in optimization considering uncertainties—An overview. Comput. Methods Appl. Mech. Eng..

[7] Vo-Duy T., Duong-Gia D., Ho-Huu V., Nguyen-Thoi T. (2020). An Effective Couple Method for Reliability-Based Multi-Objective Optimization of Truss Structures with Static and Dynamic Constraints. Int. J. Comput. Methods.

[8] Kaveh A., Biabani Hamedani K., Kamalinejad M. (2021). Set theoretical variants of optimization algorithms for system reliability-based design of truss structures. Period. Polytech. Civ. Eng..

[9] Ni P., Li J., Hao H., Yan W., Du X., Zhou H. (2020). Reliability analysis and design optimization of nonlinear structures. Reliab. Eng. Syst. Saf..

[10] Duan Z., Jung Y., Yan J., Lee I. (2020). Reliability-based multi-scale design optimization of composite frames considering structural compliance and manufacturing constraints. Struct. Multidiscipl. Optim..

[11] Tsompanakis Y., Papadrakakis M. (2004). Large-scale reliability based structural optimization. Struct. Multidisc. Optim..

[12] Mróz Z., Haftka R.T. (1994). Design sensitivity analysis of non-linear structures in regular and critical states. Int. J. Solids Struct..

[13] Frangopol D.M., Hendawi S. (1994). Incorporation of corrosion effects in reliability-based optimization of composite hybrid plate girders. Struct. Saf..

[14] Xiao M., Zhang J., Gao L. (2020). A system active learning Kriging method for system reliability-based design optimization with a multiple response model. Reliab. Eng. Syst. Saf..

[15] Meng Z., Li G., Wang X., Sait S.M., Yıldız A.R. (2021). A Comparative Study of Metaheuristic Algorithms for Reliability-Based Design Optimization Problems. Arch. Comput. Methods Eng..

[16] Wang Y., Zhou X., Wang H., Kong D., Xu S. (2022). Stochastic constitutive model of structural steel based on random field of corrosion depth. Case Stud. Constr. Mater..

[17] Di Sarno L., Majidian A., Karagiannakis G. (2021). The Effect of Atmospheric Corrosion on Steel Structures: A State-of-the-Art and Case-Study. Bldgs.

[18] Han X., Yang D.Y., Frangopol M. (2021). Optimum maintenance of deteriorated steel bridges using corrosion resistant steel based on system reliability and life-cycle cost. Eng. Struct..

[19] García-Segura T., Penadés-Plà V., Yepes V. (2018). Sustainable bridge design by metamodel-assisted multi-objective optimization and decision-making under uncertainty. J. Clean. Prod..

[20] Guo Y.L., Chen H., Pi Y.L., Bradford M.A. (2016). In-plane strength of steel arches with a sinusoidal corrugated web under a full-span uniform vertical load: Experimental and numerical investigations. Eng. Struct..

[21] He J., Wang S., Liu Y., Dai L., Lyu Z., Li C., Xin H., Tan C. (2021). The development of composite bridges with corrugated steel webs in China. Proc. Inst. Civ. Eng. Bridge Eng..

[22] Li Y., Zhang W., Zhou Q., Qi X., Widera G.E. (2000). Buckling strength analysis of the web of a WCW H-beam: Part 2: Development and research on H-beams with Wholly Corrugated Webs (WCW). J. Mater. Process. Technol..

[23] Zirakian T., Lim J.B.P., Hajsadeghi M., Bahrebar M. (2016). Structural performance of corrugated web steel coupling beams. Proc. Inst. Civ. Eng. Struct. Build..

[24] Pasternak H., Kubieniec G. (2010). Plate girders with corrugated webs. J. Civ. Eng..

[25] Wang S., Liu Y., He J., Xin H., Yao H. (2019). Experimental study on cyclic behavior of composite beam with corrugated steel web considering different shear-span ratio. Eng. Struct..

[26] Elgaaly M., Seshadri A., Hamilton R.W. (1997). Bending strength of steel beams with corrugated webs. J. Struct. Eng. ASCE.

[27] Sayed-Ahmed E.Y. (2005). Plate girders with corrugated steel webs. Eng. J..

[28] Kövesdi B., Jáger B., Dunai L. (2016). Bending and shear interaction behavior of girders with trapezoidally corrugated webs. J. Constr. Steel. Res..

[29] Kövesdi B., Dunai L., Kuhlmann U. (2012). Interacting stability behaviour of steel I-girders with corrugated webs. Thin Wall. Struct..

[30] Zhou M., Yang D., Zhang J., An L. (2017). Stress analysis of linear elastic non-prismatic beams with corrugated steel webs. Thin Wall. Struct..

[31] Lopes G.C., Couto C., Real P.V., Lopes N. (2017). Elastic critical moment of beams with sinusoidally corrugated webs. J. Constr. Steel Res..

[32] Elkawas A.A., Hassanein M.F., El Hadidy A.M., El-Boghdadi M.H., Elchalakani M. (2021). Behaviour of corrugated web girders subjected to lateral-torsional buckling: Experimental tests and numerical modelling. Structures.

[33] Hassanein M.F., Elkawas A.A., El Hadidy A.M., Elchalakani M. (2017). Shear analysis and design of high-strength steel corrugated web girders for bridge design. Eng. Struct..

[34] Wang P.Y., Garlock M.E.M., Zoli T.P., Quiel S.E. (2021). Low-frequency sinusoids for enhanced shear buckling performance of thin plates. J. Constr. Steel Res..

[35] Sokołowski D., Kamiński M. (2021). FEM Study of a Steel Corrugated Web Plate Girder Subjected to Fire. Int. J. Appl. Mech. Eng..

[36] Pimenta R.J., Queiroz G., Diniz S.M.C. (2015). Reliability-based design recommendations for sinusoidal-web beams subjected to lateral-torsional buckling. Eng. Struct..

[37] Sokołowski D., Kamiński M. (2015). Reliability analysis of the corrugated I-beam girder with ribs. Eng. Comput. Mech..

[38] Shon S., Yoo M., Kang J., Lee S. (2015). Minimum Weight Design of Sinusoidal Corrugated Web Beam using Differential Evolution Algorithm. Int. J. Steel Struct..

[39] (2004). Basis of Structural Design.

[40] Papadrakakis M., Lagaros N.D., Plevris V. (2005). Design optimization of steel structures considering uncertainties. Eng. Struct..

[41] Computer Algebra System MAPLE 2018 Documentation edn., 2019. https://fr.maplesoft.com/documentation_center/index.aspx.

[42] Kamiński M. (2013). The Stochastic Perturbation Method for Computational Mechanics.

[43] Melchers R. (2002). Structural Reliability Analysis and Prediction.

[44] (2019). Dassault Systèmes, version 2020x edn.

